# Fetal growth restriction exhibits various mTOR signaling in different regions of mouse placentas with altered lipid metabolism

**DOI:** 10.1007/s10565-024-09855-8

**Published:** 2024-03-07

**Authors:** Jie Dong, Qian Xu, Chenxi Qian, Lu Wang, Alison DiSciullo, Jun Lei, Hui Lei, Song Yan, Jingjing Wang, Ni Jin, Yujing Xiong, Jianhua Zhang, Irina Burd, Xiaohong Wang

**Affiliations:** 1 Department of Obstetrics and Gynecology, Tangdu Hospital, Air Force Medical University, NO. 569, Xinsi Road, Baqiao District, Xi’an, 710038 Shaanxi Province China; 2 Department of Obstetrics, Gynecology and Reproductive Sciences, University of Maryland, 22 S. Greene Street, Suite P6H302, Baltimore, MD 21201 USA; 3 Department of Obstetrics and Gynecology, The Second Affiliated Hospital of Xi’an Medical University, Xi’an, 710038 Shaanxi Province China

**Keywords:** Fetal growth restriction, Placenta, mTOR signaling, Lipid metabolism, Maternal exercise

## Abstract

Fetal growth restriction (FGR) is a common complication of pregnancy and can have significant impact on obstetric and neonatal outcomes. Increasing evidence has shown that the inhibited mechanistic target of rapamycin (mTOR) signaling in placenta is associated with FGR. However, interpretation of existing research is limited due to inconsistent methodologies and varying understanding of the mechanism by which mTOR activity contributes to FGR. Hereby, we have demonstrated that different anatomic regions of human and mouse placentas exhibited different levels of mTOR activity in normal compared to FGR pregnancies. When using the rapamycin-induced FGR mouse model, we found that placentas of FGR pregnancies exhibited abnormal morphological changes and reduced mTOR activity in the decidual-junctional layer. Using transcriptomics and lipidomics, we revealed that lipid and energy metabolism was significantly disrupted in the placentas of FGR mice. Finally, we demonstrated that maternal physical exercise during gestation in our FGR mouse model was associated with increased fetal and placental weight as well as increased placental mTOR activity and lipid metabolism. Collectively, our data indicate that the inhibited placental mTOR signaling contributes to FGR with altered lipid metabolism in mouse placentas, and maternal exercise could be an effective method to reduce the occurrence of FGR or alleviate the adverse outcomes associated with FGR.

**Graphical Abstract:**

- Human and mouse placentas have different mTOR signaling activities in different anatomic regions in normal and FGR pregnancies.
- Pregnant mice with FGR induced by rapamycin show smaller placentas, decreased mTOR activity in DJ layer of placenta and altered lipid metabolism.
- Maternal exercise partially alleviates the abnormal outcomes of FGR model.

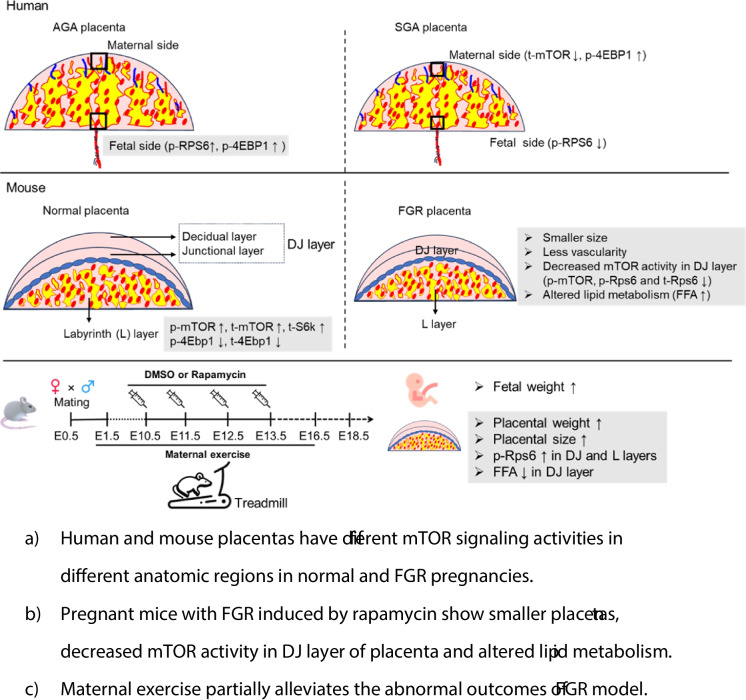

**Supplementary Information:**

The online version contains supplementary material available at 10.1007/s10565-024-09855-8.

## Introduction

Fetal growth restriction (FGR) is a commonly encountered complication during human pregnancy and is frequently associated with low birth weight or small-for- gestational-age (SGA) neonates. The global prevalence of FGR ranges between 3 to 7% of all pregnancies (Chew and Verma 2022), yet developing countries experience a strikingly higher incidence of approximately 20% among live births (Lee et al. 2017). FGR can be associated with significant perinatal morbidity and mortality as well as adverse long-term developmental and neurologic outcomes (von Beckerath et al. 2013). The underlying etiology of FGR can be heterogenous and associated with maternal conditions (e.g., malnutrition, hypertension, preeclampsia, environmental toxins), fetal anomalies (e.g., genetic aberrations, multiple fetuses), or placental dysfunction (Malhotra et al. 2019). Inadequate placental perfusion is thought to be responsible in the majority of FGR cases (Burton and Jauniaux 2018).

The placenta is a vital organ of pregnancy and proper function is required to ensure adequate fetal growth and development. The placenta acts as a maternal–fetal immune barrier and is responsible for the transfer of nutrients and oxygen from the maternal circulation to the developing fetus while also secreting hormones and cytokines (Mess and Carter 2007). In response to suboptimal uterine environments (including gestational diabetes, malnutrition, inflammation, or infection) the placenta exhibits a remarkable adaptive ability to regulate fetal growth (Sferruzzi-Perri and Camm 2016). However, when placental function becomes severely compromised due to maternal or genetic conditions, significant fetal effects can be observed, including the development of FGR. Nevertheless, the specific molecular mechanisms underlying the relationship between abnormal placental function and FGR remain inadequately understood.

The mTOR signaling pathway is critical in the regulation of cellular metabolism, growth, and survival, responding to cues from nutrient availability, energy levels, and growth factors (Saxton and Sabatini 2017). Two distinct forms of mTOR exist, mTORC1 and mTORC2. mTORC1 governs cellular growth and metabolism via the acceleration of protein, lipid, and nucleic acid synthesis, while mTORC2 is primarily implicated in cell proliferation and survival (Saxton and Sabatini 2017). A salient function of mTORC1 is its ability to promote protein translation via the phosphorylation of downstream effector molecules, including ribosomal S6 kinase (S6K) and eukaryotic translation initiation factor 4E binding protein 1 (4EBP1) (Burnett et al. 1998). Notably, the initiation and elongation of mTORC1-dependent protein translation hinges on the phosphorylation of ribosomal protein S6 (RPS6), which serves as a direct downstream effector of S6K (Holz et al. 2005). Consequently, the phosphorylation levels of mTOR, S6K, RPS6, and 4EBP1 often represent useful indices of mTOR signaling activity (Dong et al. 2020).

Increasing evidence indicates that the placental mTOR signaling pathway plays a crucial role in balancing maternal nutrient availability to support fetal growth (Dong et al. 2020; Roos et al. 2009). However, the underlying mechanism by which reduced mTOR activity in the placenta contributes to FGR remains poorly understood. In fact, despite the majority of studies showing inhibition of mTOR activity in FGR placentas, inconsistencies remain which hinder the ability to clearly understand the relationship between placental mTOR activity and fetal growth (Dong et al. 2020). The heterogeneity in research results among studies may be due to not using identical methods to measure mTOR activity, or the potential impact of different cell types (or anatomic regions) on mTOR signaling in the placenta. Our previous research with mouse placentas demonstrated that different anatomical regions exhibit varying mTOR activity, as indicated by downstream effectors, when exposed to maternal inflammation (Dong et al. 2019). Based on these findings, we proposed that key molecules involved in mTOR signaling, such as mTOR, S6K, RPS6, and 4EBP1, may exhibit distinct activities across different placental regions.

In the present study, we conducted a comprehensive investigation of mTOR signaling activity in various anatomic layers of human and mouse placentas under normal conditions. We then examined differences in mTOR signaling in various placental regions in human placentas from SGA pregnancies and mouse placentas using the rapamycin-induced FGR model (rapamycin being a known inhibitor of mTOR activity). We utilized transcriptomic and lipidomic analyses to understand the molecular mechanisms underlying the role of mTOR signaling in FGR. Lastly, since we observed an increased high fat state in mouse placenta of rapamycin-induced FGR in mice, we investigated the potential of maternal exercise as a moderator to promote fetal growth. The objective of our study was to elucidate the precise mechanism by which mTOR signaling may contribute to FGR, therefore identifying interventional targets in the prevention of FGR.

## Materials and Methods

### Sample collection

#### Human placenta and umbilical cord blood collection

All placentas were collected immediately after delivery of full-term newborns by elective cesarean section without pregnancy-related complications (such as gestational hypertension, diabetes, placenta abruption etc.). Neonates were classified as either SGA, if the birth weight was below the 10th percentiles for gestational age, or appropriate for gestational age (AGA), if the birth weight was between the 10th to 90th percentiles for gestational age. Placental tissue (~ 0.5 cm thick) was harvested from both the fetal and maternal placental sides, near the umbilical cord insertion site. The biopsies were washed in cold PBS to remove blood, immediately frozen in liquid nitrogen, and stored at -80℃ for subsequent use.

#### Mouse sample preparation

All animal experiment procedures were conducted in Air Force Medical University according to the Animal Protection Guidelines of Air Force Medical University (SYXK2019-001). Four-week-old ICR female mice and adult ICR male mice were obtained from Beijing HFK Bioscience Company (Beijing, China). All mice were housed at 22–24 ℃ with constant light between 8 am and 8 pm and had unrestricted access to food and water. The food used for raising all mice was common dietary formula (20% protein + 4% fat + 5% fibre) and purchased from Beijing HFK Bioscience Company (Cat. #1025). Female (6 weeks old) and male mice (6–10 weeks old) were caged together at ratio of 1:1 to facilitate mating. Embryonic day 0.5 (E0.5) was defined based on identification of a vaginal plug the day after mating. Pregnant mice were sacrificed by cervical dislocation. We collected mouse placentas at E14.5 (a time point for mouse placenta maturation) or E18.5 and performed tissue separation with the aid of a dissecting microscope. To obtain the different regions of mouse placentas, we transected the mouse placenta in half, then divided one half to decidual-junctional (DJ) layer and labyrinth (L) layer according to placental structure. The isolated samples were preserved in liquid nitrogen and stored at -80℃. A subset of intact samples was fixed in 4% paraformaldehyde to facilitate histological analysis.

#### Mouse model of FGR

The FGR mouse model was developed using pregnant 6-week-old ICR female mice. They were subjected to daily intraperitoneal injection of either 0.01 mg or 0.02 mg rapamycin (Selleck, #1039, USA) or Dimethyl sulfoxide (DMSO, Sigma, #D8418, USA) diluted in phosphate-buffered saline (PBS) from E10.5 to E13.5. Pregnant mice were sacrificed by cervical dislocation at E14.5 or E18.5. At that time maternal blood samples were collected and fetal and placental data were recorded. The same procedures used for placental dissection were performed as the above mentioned.

#### Maternal exercise

To explore the effects of exercise during pregnancy on fetal weight in the rapamycin-induced FGR model pregnant mice were subjected to flat treadmill exercise. The running parameters were set according to the method published by Son et al. (2019). In brief, pregnant mice were separated into three stages, E1.5 – E7.5, E8.5 – E14.5, and E15.5 – E16.5. The exercise protocol consisted of three phases: warming up (5 m/min for 10 min), exercise (10 – 14 m/min for 40 min), and cooling down (5 m/min for 10 min), which were performed at the same time every morning. The treadmill speed during the exercise phase was set to 11, 14, and 12.5 m/min for E1.5 – E7.5, E8.5 – E14.5 and E15.5 – E16.5, respectively. Mice were not included in running exercise between E16.5 and E18.5 to avoid possible acute effects of this activity on sample collection at E18.5.

### Histological analysis of mouse placenta

Mouse placentas were fixed in paraformaldehyde overnight before being processed and embedded into paraffin wax for histological staining. Each sample was sectioned at 6 μm thickness perpendicular to the chorionic plate (Leica RM2235, Germany). Slides were then stained with hematoxylin & eosin (HE). All stained slides were covered by dibutyl phthalate polystyrene xylene mounting medium (Sigma) and scanned (Olympus VS120, Japan) to assess placental thickness and distinguish DJ and L layers. For immunofluorescence (IF) staining, the slides were incubated in heated antigen unmasking solution (Vector Laboratories, USA) for antigen retrieval, and the remaining steps were similar to those previously described (Dong et al. 2019). The primary antibodies for IF included Tpbpa (a marker for spongiotrophoblast cells, Abcam, #ab104401), Vimentin (a marker for interstitial cell, Abcam, #ab8978) and CD31 (a marker for endothelial cells, Abcam, #ab182981). The secondary antibodies included Goat anti-rabbit IgG Alexa Fluor 568 or 488 and Goat anti-mouse IgG Alexa Fluor 568 (Life Technologies). All IF slides were finally covered using Fluromount-G (eBioscience). Images were photographed and scanned using the Olympus VS120. We analyzed the intensity and density of positive regions on IF slides by Image J software (NIH, Bethesda, USA).

### Western blot (WB)

For protein sample extraction, placental tissues were homogenized on ice in Protein Extraction Reagent (Thermo, #78510) with protease inhibitor (MCE, #HT-K0011) and phosphatase inhibitor (Roche, #04906837001). The homogenized specimens were then placed on ice for 20 min and centrifuged at 14,000 rpm for 20 min at 4 °C, the resultant supernatant was isolated. We used Coomassie Staining solution (Beyotime, #P0017F, China) to determine the quality and loading volume of protein samples. Total protein was separated by sodium dodecyl sulfate–polyacrylamide gel electrophoresis using 4–20% gels (Biorad, #4561096, USA) and then transferred onto PVDF membranes using Trans-Blot Turbo RTA Transfer Kit (Biorad, #1704272) via a semidry transfer device (Bio-Rad). Membranes were blocked with 5% bovine serum albumin (MP, #FC0077) in Tris-buffered saline containing 0.1% Tween-20 (TBST) for 30 min at room temperature, incubated with primary antibodies in 5% BSA at 4 °C overnight, then washed with TBST three times (10 min each). The secondary antibody was HRP-labelled goat anti-rabbit IgG. Protein bands were visualized using an Immobilon Chemiluminescence Reagent (Millipore, #P90720) and recorded by a ChemiDox Gel imaging system (Biorad). The primary antibodies (diluted at 1:1000) used were Tpbpa (Abcam, #ab104401), phospho-mTOR Ser2448 (p-mTOR, CST, #5536), total mTOR (t-mTOR, CST, #2983), total S6K (t-S6K, CST, #2708), phospho-RPS6 Ser235/6 (p-RPS6, CST, #4858), total RPS6 (t-RPS6, CST, #2217), phospho-4EBP1 Thr37/46 (p-4EBP1, CST, #2855), and total 4EBP1 (t-4Ebp1, CST, #9644). Although the level of phospho-S6K Thr389 (p-S6K, CST, #9234) was also detected, difficulty in identifying specific bands in both mouse and human placentas made it impossible to accurately analyze placental p-S6K and therefore was not analyzed. To determine the oxidative phosphorylation (OXPHOS) level, we utilized an antibody kit (Thermo, #45–8099) suitable for detecting the relative levels of the five OXPHOS complexes (CI-V) by WB. Immunological blots corresponding to CI, CII and CV were observed. For analyzing the target protein levels, total protein shown by Coomassie staining was used as the reference and Image J software was applied to calculate protein band gray values.

### mRNA sequencing

Total RNA was extracted from mouse placentas using RNeasy mini kits (Qiagen, Germany) according to the operation manual. The integrity of RNA was identified by Agilent Bioanalyzer 2100, and the concentration and purity were measured by Qubit®3.0 Fluorometer (Life Technologies, USA) and Nanodrop One spectrophotometer (Thermo). The mRNA sequencing libraries were constructed using NEBNext® UltraTM RNA Library Prep Kits for Illumina NovaSeq 6000 (USA) following the manufacturer’s protocol. Paired-end mRNA libraries were constructed by using the TruSeq™ RNA Sample Preparation Kits (Illumina, USA) following the guidebook. Briefly, the poly-A containing mRNA molecules were purified using poly-T oligo-attached magnetic beads. After purification, the mRNAs were fragmented into small pieces using divalent cations under 94℃ for 8 min. The cleaved RNA fragments were copied into first strand cDNA using reverse transcriptase and random primers. This was followed by second strand cDNA synthesis using DNA Polymerase I and RNase H. These cDNA fragments then went through an end repair process, the addition of a single A’ base, and ligation of the adapters. The products were then purified and enriched by PCR to create the final cDNA library. Purified libraries were quantified by Qubit® 2.0 Fluorometer (Life Technologies, USA) and validated by Agilent 2100 bioanalyzer (Agilent Technologies, USA) to confirm the insert size and calculate the mole concentration. Clusters were generated by cBot with the library diluted to 10 pM then sequenced on the Illumina NovaSeq 6000 (Illumina, USA). Differential expression analysis for mRNA was performed using R package. The thresholds of the differentially expressed (DE) mRNA were set as |log2(fold change)|≥ 1.5 and *p*-value < 0.05, and retained for further analysis. Functional enriched signaling pathways were analyzed for the overlapped DE mRNAs. Gene Ontology (GO) biological process, Kyoto Encyclopedia of Genes and Genomes (KEGG) Pathway, Reactome Gene Sets and WikiPathway terms were searched and enriched by Metascape.

### Real-time quantitative polymerase chain reaction (RT-qPCR)

Total RNA of mouse placentas was extracted using Trizol reagent (Invitrogen, #15,596,026) according to the instructions and added DNaseI to avoid DNA disruption. The purity and concentration of RNA were determined by a Nanodrop 2000 spectrophotometer (Thermo). A total of 0.5 µg of total RNA was converted to cDNA using a commercial reverse transcription kit (Takara, #6210A). Each cDNA sample was diluted tenfold, followed with reaction system construction. The reaction mixture for RT-qPCR consisted of 2 µL of diluted cDNA, 0.8 µL of each primer (0.1 μM), 10 µL of SYBR premix Ex Taq™ II (Takara, #RR42LR) and 6.4 µL of nuclease-free water. RT-qPCR amplification was performed under the following steps: 95 °C for 3 min, 40 cycles at 95 °C for 15 s, 58 °C for 30 s and 72 °C for 2 min. All samples were run in duplicate. The expression levels of target genes were normalized by housekeeping gene *β-Actin*. The primer sequences were obtained from Primer Bank, provided from Sangon biotech company (Shanghai, China) and shown in Supplementary Table 4. The ΔΔCt method was used for analyzing the mRNA relative expressions.

### Lipidomics analysis

Lipids were extracted from approximately 30 mg of frozen placental tissue using a modified version of the Bligh and Dyer's method as described by Lam et al. (2022). Briefly, tissues were homogenized in 750 µL of chloroform, methanol, and MilliQ H_2_O solution (3:6:1, v/v/v). The homogenate was then incubated and centrifuged at 1500 rpm for 1 h at 4℃. Phase separation was then induced by adding 350 µL of deionized water and 250 µL of chloroform. The samples were then centrifuged and the lower organic phase containing lipids was extracted.

Lipidomic analyses were conducted at LipidALL Technologies using a Shimadzu Nexera 20-AD coupled with Sciex QTRAP 6500 PLUS. Separation of individual lipid classes of polar lipids by normal phase (NP)-HPLC was carried out using a TUP-HB silica column (i.d. 150 × 2.1 mm, 3 µm) with the following conditions: mobile phase A (chloroform, methanol, ammonium hydroxide, 89.5:10:0.5) and mobile phase B (chloroform, methanol, ammonium hydroxide, water, 55:39:0.5:5.5). Multiple reaction monitoring (MRM) transitions were set up for comparative analysis of various polar lipids. Individual lipid species were quantified by referencing to spiked internal standards. Free fatty acids were quantitated using d31-16:0 (Sigma) and d8-20:4 (Cayman Chemicals). Glycerol lipids including diacylglycerols (DAG) and triacylglycerols (TAG) were quantified using a modified version of reverse phase HPLC/MRM. Separation of neutral lipids were achieved on a Phenomenex Kinetex-C18 column (i.d. 4.6 × 100 mm, 2.6 µm) using an isocratic mobile phase containing chloroform, methanol, 0.1 M ammonium acetate 100:100:4 (v/v/v) at a flow rate of 300 µL for 10 min. Levels of short-, medium-, and long-chain TAGs were calculated by referencing to spiked internal standards of TAG(14:0)3-d5, TAG(16:0)3-d5 and TAG(18:0)3-d5 obtained from CDN isotopes, respectively. DAGs were quantified using d5-DAG17:0/17:0 and d5-DAG18:1/18:1 as the internal references (Avanti Polar Lipids). Free cholesterols (Cho) and cholesteryl esters (CE) were analyzed under atmospheric pressure chemical ionization mode on a Jasper HPLC coupled to Sciex 4500 MD as reported by Shui et al. (2011), using d6-cholesterol and d6-C18:0 CE as internal standards.

### Assay of (free fatty acid) FFA, TAG and Cho

For measuring FFA, TAG, and Cho concentrations in mouse placentas, we utilized the relevant commercial kits purchased from Solarbio (Beijing, China), including FFA detection kit (Cat: #BC0590), TAG detection kit (Cat: #BC0620), total Cho detection kit (Cat: #BC1980), and free Cho detection kit (Cat: #BC1890). The spectrophotometry testing procedures were in accordance with the instructions. The level of Cho Easter was calculated by subtraction of free Cho from total Cho.

### Statistical analysis

Data processing and graphic production were achieved using Graphpad Prism software 8. Continuous variables were presented as mean ± standard deviation. For two-group comparison, the student’s t-test was used for parametric data while the Mann–Whitney test for nonparametric data. For multiple group analysis, one-way ANOVA was employed and Tukey’s test was for subsequent pairwise comparisons. A *p*-value < 0.05 was considered significant.

## Results

### Differential expression of key molecules in mTOR pathway in different anatomic regions of human placentas

**Fig. 1:**
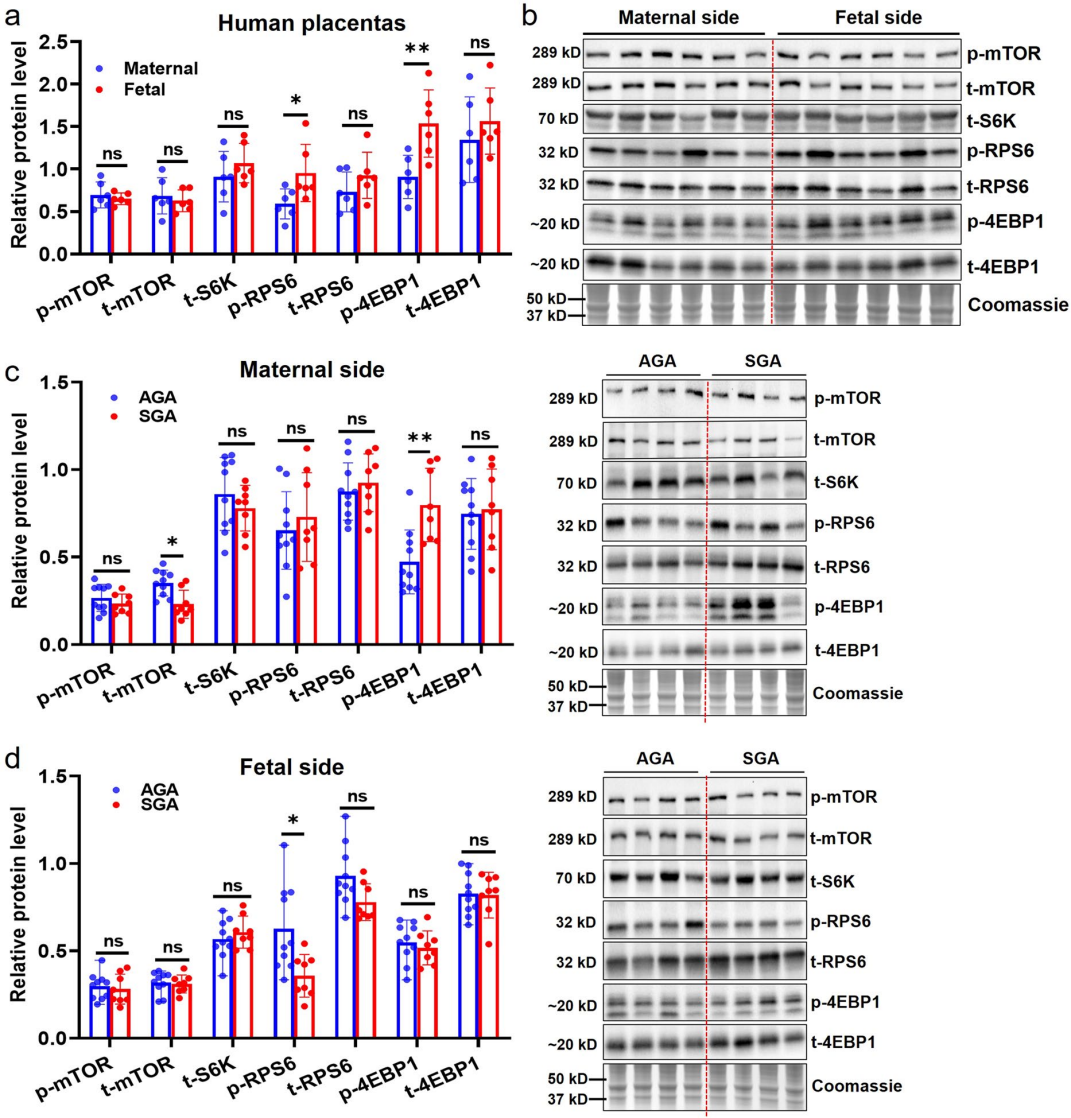
The protein levels of the key genes associated with mTOR signaling in human full-term placentas. (**a**): The comparison of mTOR-related protein levels (t-mTOR, t-S6K, t-RPS6 and t-4EBP1) and phosphorylation activities (p-mTOR, p-RPS6 and p-4EBP1) between maternal-side and fetal-side of human placentas (*n* =6/each side). (**b**): The representative blots of mTOR-related molecules and total proteins shown by Coomassie staining in normal full-term human placentas. (**c**): The comparison of mTOR-related protein levels and phosphorylation activities in maternal side of human placentas between AGA (*n* =10) and SGA (*n* =8) group, and the representative blots. (**d**): The comparison of mTOR-related protein levels and phosphorylation activities in fetal side of human placentas between AGA (*n* =10) and SGA (*n* =8) group, and the representative blots. Pair-wise or Student’s t-test was used for statistical analysis and *p*-value < 0.05 was significant. ns: not significant, *p < 0.05, **p < 0.01

We firstly compared the levels of the total and phosphorylated mTOR-related proteins (including mTOR, S6K, RPS6 and 4EBP1) in the placentas of normal human pregnancies (clinical information shown in Supplementary Table 1). We found that p-RPS6 and p-4EBP1 had significantly higher levels in the fetal side of placenta compared to the maternal side, and the levels of p-mTOR, t-mTOR, t-S6K, t-RPS6 and t-4EBP1 were similar between the fetal and maternal sides (Fig. 1a and b). The results suggested that the activities of mTOR-related proteins in the fetal side were different from the maternal side.

Subsequently, we analyzed the changes in mTOR activity in the fetal and maternal sides of placentas from SGA compared to control, or AGA, neonates. The baseline clinical data are reported in Supplementary Table 2. Our results showed that in the maternal side of SGA placentas the expression of t-mTOR was significantly decreased while the level of p-4EBP1 was increased, compared to controls (Fig. 1c). However, in the fetal side of SGA placentas, we only observed a robust reduction in the level of p-RPS6 (Fig. 1d). These findings suggest that mTOR activity exhibits inconsistent patterns in different anatomic locations of human placentas under both normal and SGA conditions.

### Differential expression of mTOR-related proteins in different anatomic layers of mouse placentas

**Fig. 2:**
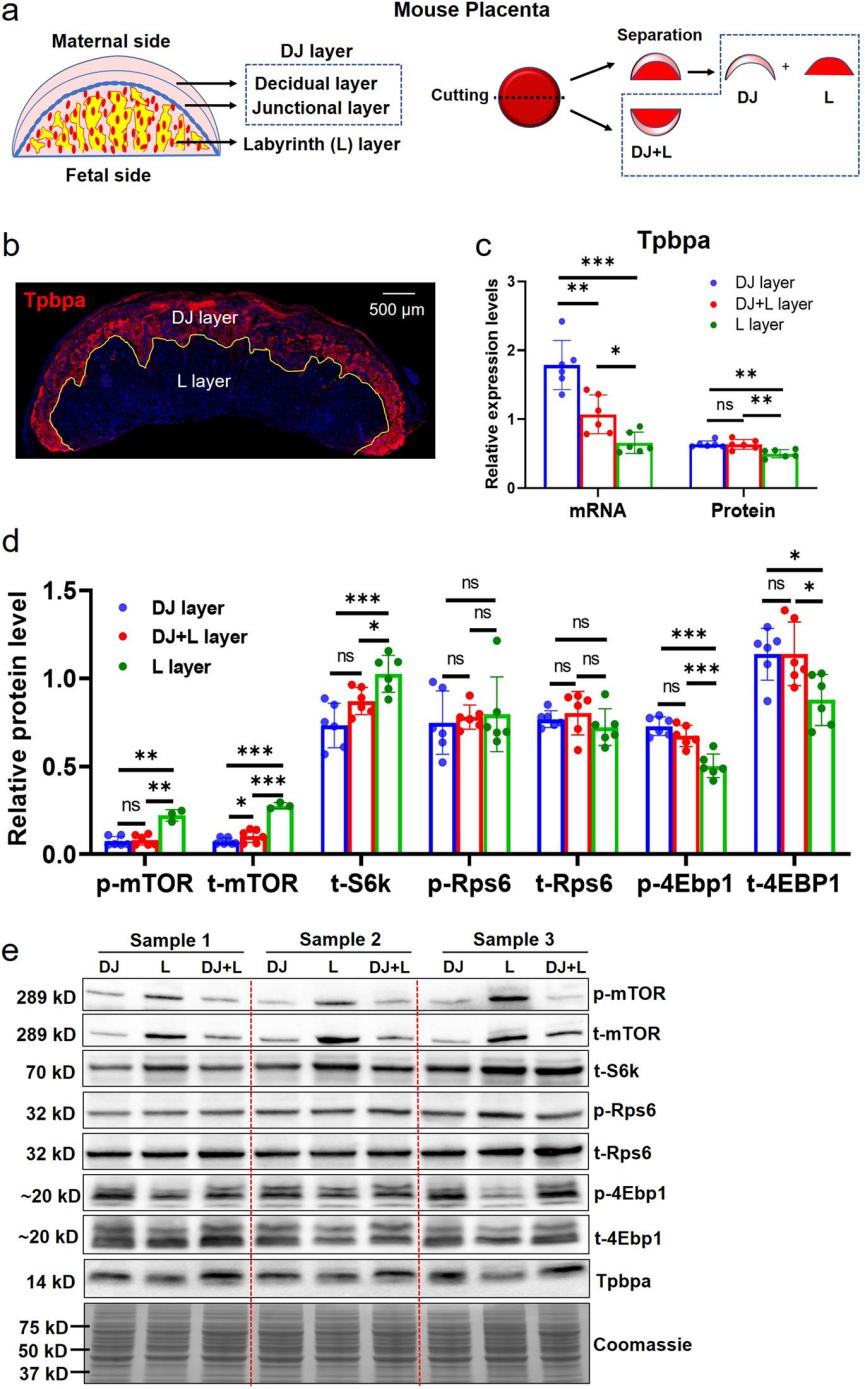
The expression levels of Tpbpa, mTOR, S6k, Rps6 and 4Ebp1 in different anatomic regions of mouse placentas at E14.5. (**a**): The separation of mouse placenta based on its structure layers. One mouse placenta was divided into decidual and junctional (DJ) layer, labyrinth (L) layer and half of the whole one (DJ + L layer) for RT-qPCR and WB. (**b**): The IF staining of Tpbpa, a marker for spongiotrophoblast cells, was used to distinguish DJ and L layer. (**c**): The mRNA and protein expression of *Tpbpa* in DJ, L and DJ + L layers of mouse placentas (*n* =6 dams). (**d**): The mTOR-related protein levels and phosphorylation activities in different anatomic regions (DJ, L and DJ + L) of mouse placentas (*n* =6 dams). (**e**): The representative protein blots. One-way ANOVA test was used for the global comparison and Turkey’s test for the pairwise comparison, and *p*-value < 0.05 was significant. ns: not significant, *p<0.05, **p<0.01, ***p<0.001

As depicted in Fig. 2a, we partitioned the mouse placenta into three segments: the decidua junction (DJ) layer, the labyrinth (L) layer, and the composite DJ and L (DJ + L) layer. HE staining displayed the general structures of DJ, L and DJ + L layers, as shown in Figure S1. By means of IF staining of Tpbpa, a specific biomarker for spongiotrophoblast cells, we distinguished the DJ and L layers in the mouse placenta (Fig. 2b). The RT-qPCR and WB assays demonstrated that *Tpbpa* mRNA and protein were preferentially expressed in the DJ layer compared to the L layer, indicating the significance and purpose of our mouse placenta separation methodology for subsequent analyses (Fig. 2c and e). We assessed the protein levels and phosphorylation activities of four key mTOR-related genes (mTOR, S6k, Rps6, and 4Ebp1) in mouse placentas. Our results indicated that the levels of p-mTOR, t-mTOR, and t-S6k were significantly lower in the DJ layer than in the L layer, whereas the levels of p-4Ebp1 and t-4Ebp1 were markedly higher in the DJ layer than in the L layer. No differences were found in the levels of p-Rps6 and t-Rps6 between the DJ and L layers (Fig. 2d and e). Our findings demonstrate that the key molecules in mTOR signaling exhibit variable expression and phosphorylation activity across distinct anatomic regions in mouse placentas.

### Differential expression of mTOR-related proteins in different anatomic layers of rapamycin-induced FGR mouse placentas

**Fig. 3:**
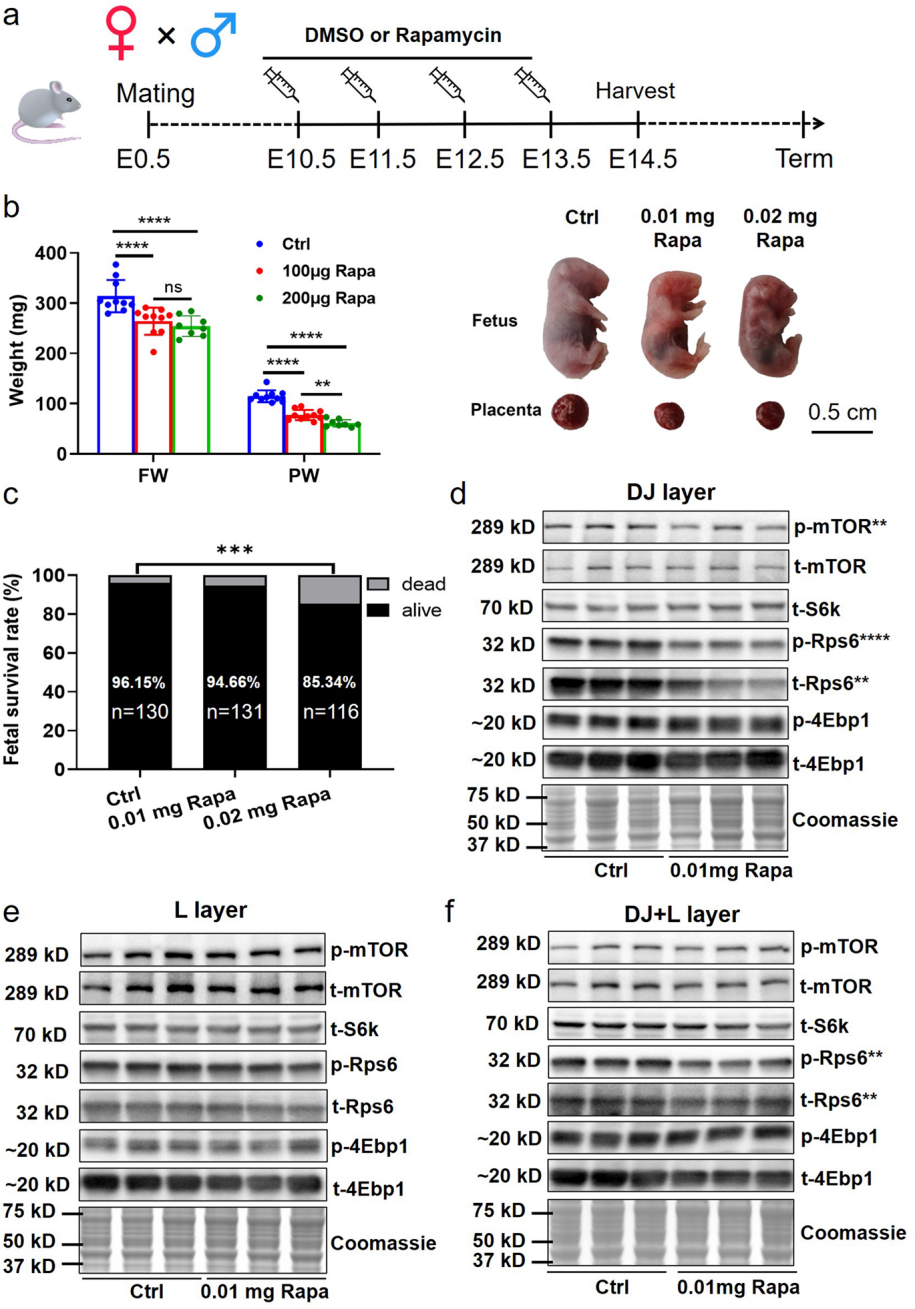
The activity changes of mTOR signaling in different regions of mouse placentas in the FGR model. (**a**): The rapamycin-induced FGR model. Pregnant mice at E10.5 received daily intraperitoneal injection with 0.01 or 0.02 mg rapamycin or DMSO per dam from E10.5 to E13.5, and sacrificed at E14.5. (**b**): The fetal weight and placental weight at E14.5 in control (Ctrl) and rapamycin (Rapa) group (*n* =8–10 dams/each group). (**c**): The survival rate of fetuses after rapamycin injection (n: the number of fetuses). (**d**): The mTOR signaling activity in DJ layer after rapamycin treatment (*n* =9 dams/each group). (e): The mTOR signaling activity in L layer after rapamycin treatment (*n* =9 dams/each group). (**f**): The mTOR signaling activity in DJ + L layer after rapamycin treatment (*n* =9 dams/each group). One-way ANOVA test or student’s t-test was used for the statistical analysis. ns: not significant, **p<0.01. ****p<0.0001

Given the differential expression and phosphorylation of key proteins in the mTOR signaling pathway across anatomical regions of normal mouse placenta, we sought to investigate whether there would be variation in these findings with FGR placentas. Utilizing the rapamycin-induced FGR mouse model (Fig. 3a), pregnant mice receiving low-dose (0.01 mg/kg) or high-dose (0.02 mg/kg) rapamycin exhibited significantly reduced fetal and placental weight at E14.5, with the survival rate of fetuses being lower in the high-dose group (Fig. 3b, c). We primarily compared control and low-dose rapamycin groups in the subsequent experiments.

To confirm the inhibitory effect of rapamycin on placental mTOR signaling in mice, we examined mTOR activity in the three parts of mouse placentas, as depicted in Fig. 2a. Our results showed that in the DJ layer the levels of p-mTOR, p-Rps6, and t-Rps6 were significantly reduced in rapamycin-treated compared to control placentas (Fig. 3d and Figure S2a-d). In contrast, no changes were observed in the levels and activities of mTOR-related targets in the L layer between the two groups (Fig. 3e and Figure S2e-h). Notably, in placentas containing both DJ and L components, there was a substantial decrease in the levels of p-Rps6 and t-Rps6 in the rapamycin group compared to control (Fig. 3f and Figure S2i-l). These data collectively suggest that FGR established by inhibiting placental mTOR signaling in our mouse model develops through variable levels of activity of key mTOR signaling molecules in different regions of the placenta.

### Histological findings in rapamycin-induced FGR mouse placentas

**Fig. 4:**
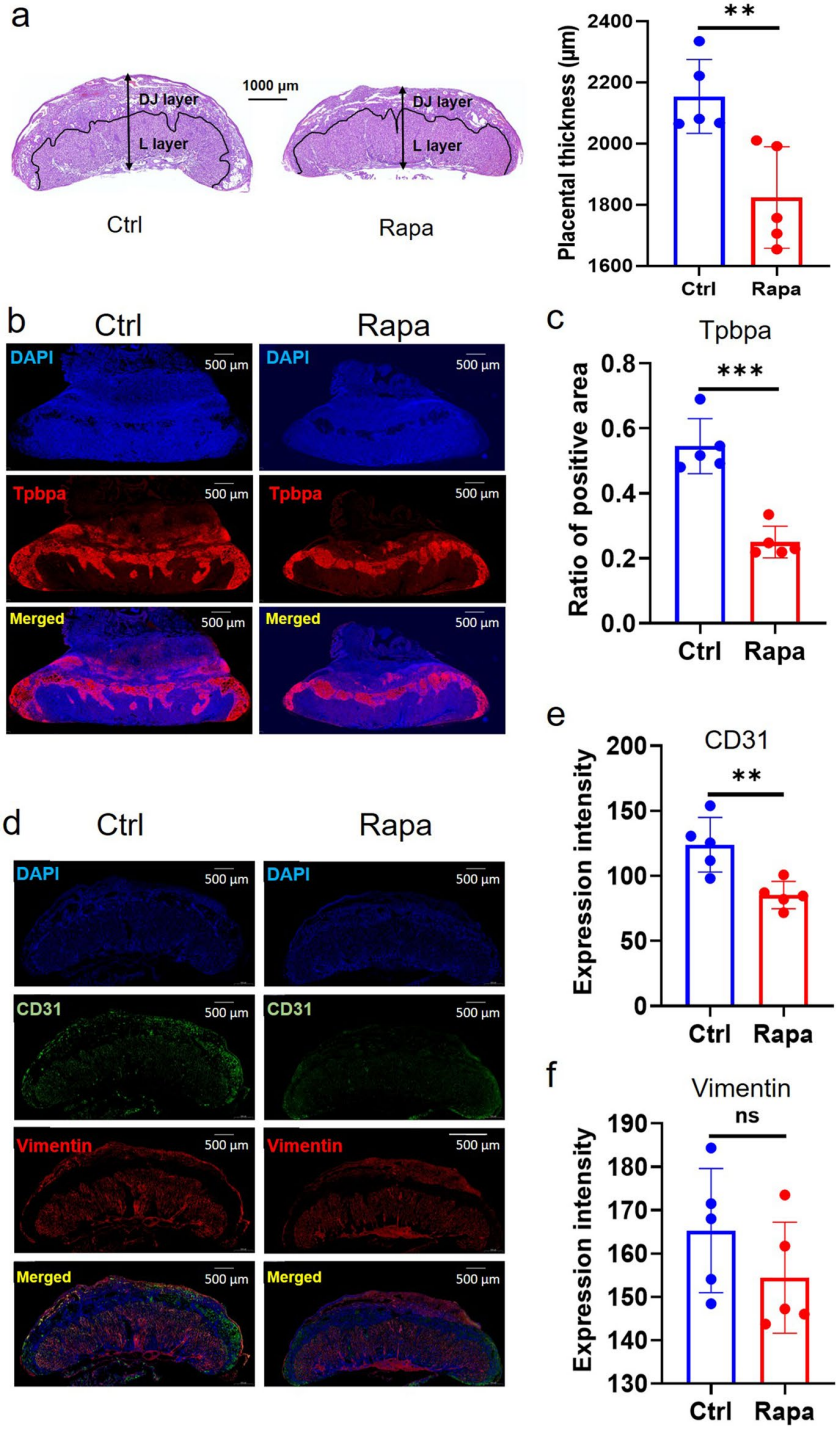
The morphological analysis of mouse placentas from Ctrl and Rapa (low-dose/ 0.01 mg Rapa) groups at E14.5. (**a**): HE staining and placental thickness (*n* =5/each group). Each placenta sample contains DJ and L layer. (**b**): IF staining of Tpbpa (red) and DAPI (blue) used for nuclear staining. (**c**): The comparison of the ratio of Tpbpa-positive area between Ctrl and Rapa placentas (*n* =5/each group). (**d**): IF staining of Vimentin (a marker for interstitial cell, red), CD31(a marker for endothelial cells, green) and DAPI (blue). (**e**): The comparison of Vimentin intensity between Ctrl and Rapa placentas (*n* =5 dams/each group). (**f**): The comparison of CD31 intensity between Ctrl and Rapa placentas (*n* =5 dams/each group). Student’s t-test was used for statistical analysis and *p*-value<0.05 was significant. ns: not significant, **p<0.01. ***p<0.001

To investigate the potential mechanisms underlying FGR induced by rapamycin treatment, we conducted morphological analyses of control and FGR placentas. HE staining revealed that FGR placentas were smaller in size and thinner than control placentas, especially in the junctional layer (Fig. 4a). To study the change of the spongiotrophoblast distribution in mouse placentas, we analyzed Tpbpa protein, which labels spongiotrophoblast cells. We observed that the Tpbpa-positive area was relatively flat in FGR placentas exposed to rapamycin, compared to the irregular shape observed in control placentas (Fig. 4b). Moreover, the ratio of Tpbpa-positive area was significantly reduced in FGR placentas (Fig. 4c). To examine the overall distribution of vascular endothelial cells and mesenchymal cells in mouse placentas, we performed IF staining using CD31and Vimentin, respectively. Our results revealed that CD31 intensity was significantly decreased in FGR placentas compared to controls, but Vimentin expression was similar (Fig. 4d**-**f, and Figure S3). Collectively, our findings suggest that the mouse placenta displays abnormal morphological characteristics in the FGR model induced by rapamycin.

### Analysis of mRNA sequencing in control and rapamycin-induced FGR mouse placentas

**Fig. 5:**
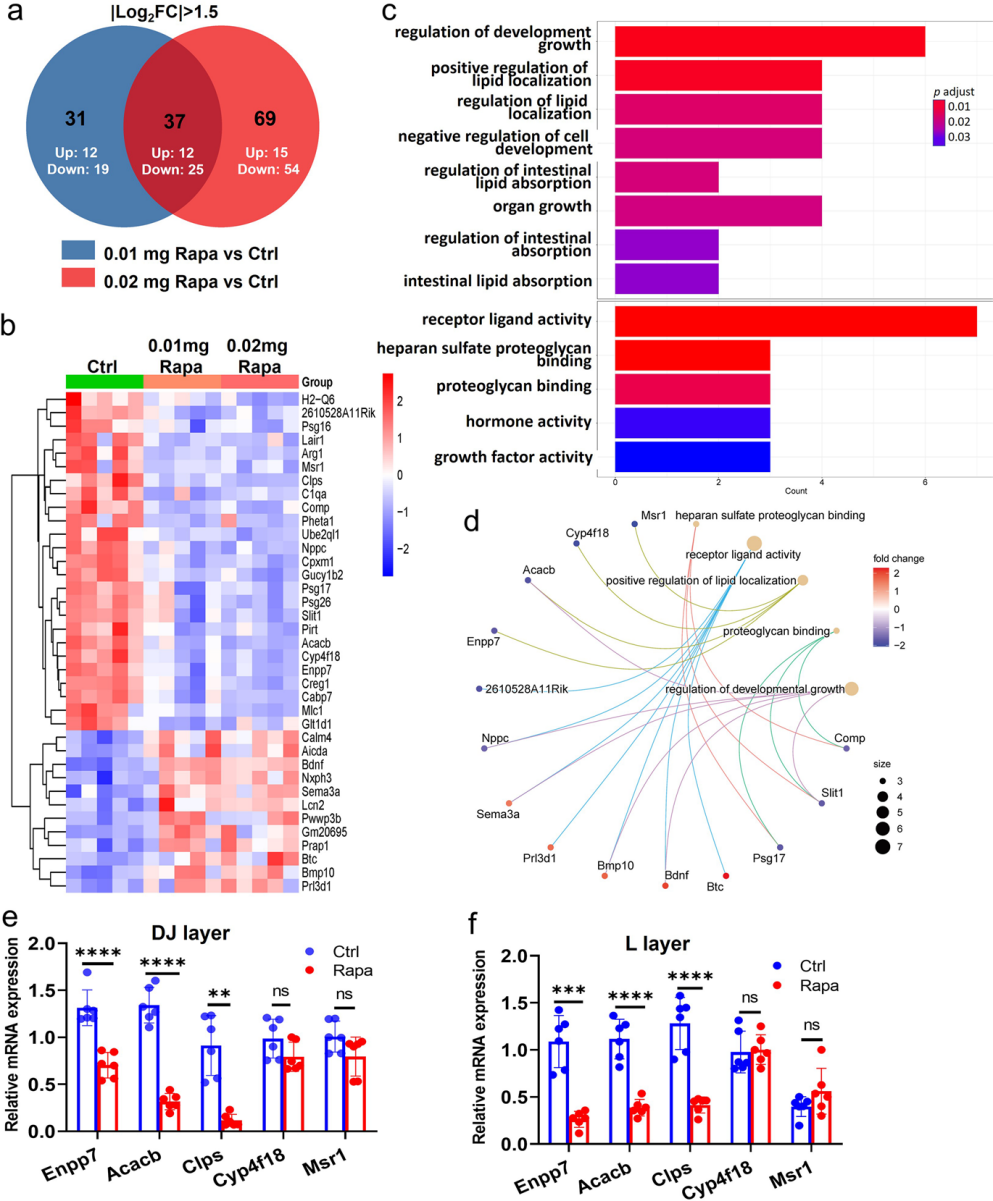
The analysis of mRNA sequencing on mouse placentas from Ctrl and Rapa fetuses at E14.5. (**a**): The number of DE mRNAs among the three groups (Ctrl, 0.01 mg Rapa and 0.02 mg Rapa, *n* =5 dams/each group). DE mRNAs were set as |Log2FC|> 1.5 and *p*<0.05. (**b**): The heatmap of DE mRNAs, showing the overlapped 37 DE mRNAs (blue: downregulation, red: upregulation). (**c**): Functional enriched signaling pathways for the overlapped DE mRNAs. (**d**): Cnetplot of KEGG enrichment analysis for common DE genes, showing enriched KEGG terms and corresponding genes. The mRNA expression identification of genes (*Enpp7*, *Acacb*, *Clps*, *Cyp4f18* and *Msr1*) associated with lipid metabolism in DJ-layer (**e**) and L-layer (**f**) of mouse placentas (*n* =6/each group) between Ctrl and 0.01 mg Rapa group. Student’s t-test was used for statistical analysis and *p*-value<0.05 was significant. ns: not significant, **p<0.01. ***p<0.001. ****p<0.0001

To explore the underlying molecular mechanisms involved in FGR induced by rapamycin, we conducted mRNA transcriptome analysis on mouse placentas from three groups: control, low-dose rapamycin, and high-dose rapamycin. Compared to control placentas, we identified 68 differentially expressed (DE) mRNA in the low-dose rapamycin group and 106 DE mRNA in the high-dose group (Fig. 5a). To further refine the DE transcripts, we focused on the 37 overlapped DE mRNA (12 upregulated and 25 downregulated) among the three groups and generated a heatmap (Fig. 5b). Subsequent enrichment analysis of the DE mRNAs revealed multiple biological processes including regulation of development growth, regulation of lipid localization, organ growth, intestinal lipid absorption, and receptor ligand activity (Fig. 5c). Cnetplot of KEGG enrichment analysis also reflected the involvement of the lipid-related pathways and corresponding genes (Fig. 5d). Given the crucial role of lipid metabolism in placental and fetal growth, we further identified the expressions of five genes (*Enpp7*, *Acacb*, *Cyp4f18*, *Msr1* and *Clps*) associated with lipid metabolism. The mRNA levels of *Enpp7*, *Acacb,* and *Clps* were significantly decreased in both the DJ and L layers of FGR placentas compared to control (consistent with the mRNA sequencing results), while the expressions of *Cyp4f18* and *Msr1* were not altered in FGR placentas (Fig. 5e and f). Collectively, these findings suggest that FGR induced by rapamycin may be attributed to the disruption of lipid metabolism within the mouse placenta.

### Analysis of lipid molecules in different anatomic layers in rapamycin-induced FGR placentas

**Fig. 6:**
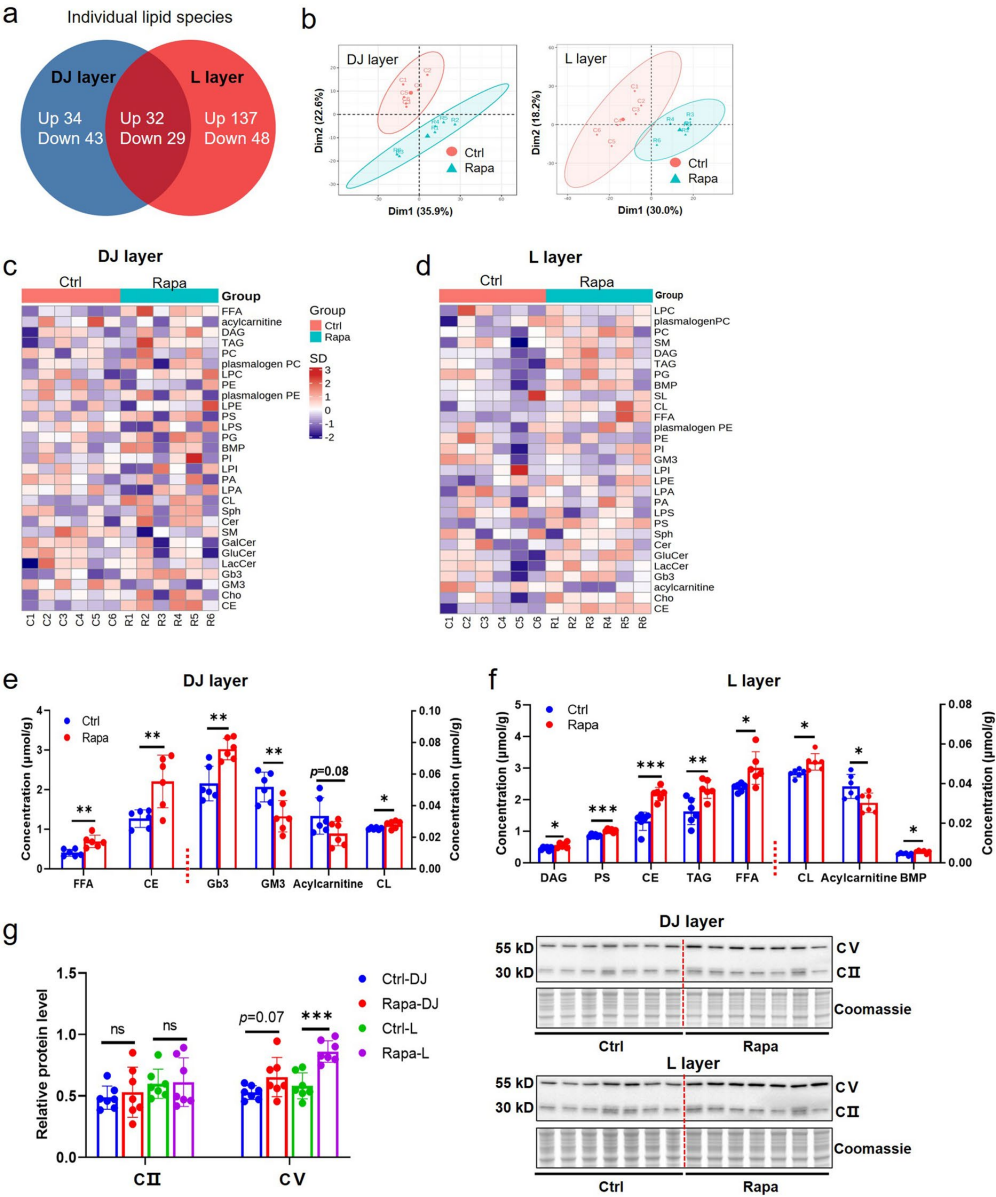
The comparison of lipid concentrations and ATP-associated protein levels between Ctrl and Rapa (low-dose/ 0.01 mg Rapa) mouse placentas at E14.5. (**a**): Principal component analysis of lipid molecule concentrations detected by lipidomics in DJ and L layer of mouse placentas (*n*=6/each group) from Ctrl and Rapa group, respectively. (**b**): Lipidomic heatmap showing the differential abundance of major lipid components in DJ layer of mouse placentas from Ctrl and Rapa group (*n* =6 dams/each group). (**c**): The heatmap showing the differential abundance of major lipid components in L layer of mouse placentas. (**d**): The comparison of multiple lipids which are significantly changed in DJ layer of Rapa placentas compared with controls (*n* =6 dams/each group). (**e**): The comparison of multiple lipids which are significantly changed in L layer of Rapa placentas comparing with controls (*n* =6 dams/each group). (**f**): The number of lipid components with significantly different levels in DJ and L layers of mouse placentas between Ctrl and Rapa group. (**g**): The expressions of ATP regulatory proteins CII and CV in DJ and L layer of mouse placentas between Ctrl and Rapa group (*n* =7 dams/each group), measured by WB. Student’s t-test was used for statistical analysis and *p*-value<0.05 was significant. ns: not significant, *p<0.05, **p<0.01, ***p<0.001

Lipids are essential for placental and fetal development and their activity may be altered in FGR placentas, as indicated by the enrichment analysis results of DE mRNAs mentioned previously. We thusly compared the lipid components between control and low-dose rapamycin groups using lipidomics analysis in both the DJ and L layers of mouse placentas. The original data are listed in Supplementary Excel 1 and 2, respectively. We quantified the comparative values of individual lipid types and found that 138 lipid molecules were changed in the DJ layer and 246 lipids changed in the L layer of FGR placentas compared to controls (Fig. 6a). A total of 61 different lipid components were simultaneously changed in the DJ and L layers of mouse placentas between the two groups (Fig. 6a, Supplementary Table 3). The principal component analysis revealed that clusters of lipid molecules in FGR placentas differed significantly from controls, regardless of the tissue origin (DJ layer or L layer) (Fig. 6b). Specifically, we analyzed 29 different types of lipids in the DJ and L layers of mouse placentas, comparing lipid content of rapamycin-induced FGR placentas to those of control placentas (Fig. 6c, d). We found that in the DJ layer four types of lipids (FFA, CE, Gb3 and CL) were substantially upregulated, while GM3 was downregulated (Fig. 6e), and in the L layer seven lipid types (DAG, PS, CE, TAG, FFA, CL and BMP) were significantly increased, whereas acylcarnitine was decreased (Fig. 6f). When further examining lipid types, we found that in general those lipids involved in energy metabolism, such as TAG and FFA, were up-regulated in FGR placentas while structural type lipids, such as CL, PE, and PS, were down-regulated (Supplementary Table 3).

Previous studies have reported that rapamycin administration can impact lipid concentrations in circulating blood (Morrisett et al. 2002; Framarino-dei-Malatesta et al. 2014). We measured the serum concentration of the three major lipids FFA, TAG, and Cho in pregnant mice and did not observe a difference between control and rapamycin groups (Figure S4 a-c). Our data suggests that FGR affects the levels of multiple lipids in mouse placentas without impacting maternal circulating lipids.

To further understand the potential mechanism underlying the lipid changes observed in FGR placentas, we assessed the mRNA levels of several genes involved in lipid oxidation (*Cpt1a*, *Cpt1b*, *Cpt1c*, *Acaca* and *Acacb*), lipogenesis (*Fasn*), lipid transport (*Slc27a2*, *Slc27a4*, *Slc27a6* and *Cd36*), and relevant regulation factors (*Srebf1*, *Srebf2*, *Soat1*, *Soat2*, *Ppar-α*, *Ppar-β/δ* and *Ppar-γ*). The results showed that in the DJ layer the most investigated genes (including *Acaca*, *Fasn*, *Slc27a6*, *Srebf1*, *Srebf2*, *Soat1*, *Soat2*, *Ppar-β/δ* and *Ppar-γ*) showed lower mRNA levels in FGR placentas than in controls (Figure S5a). Similarly, in the L layer, several genes (including *Cpt1a*, *Cpt1b*, *Fasn*, *Slc27a6*, *Cd36*, Srebf2, Soat1 and *Ppar-β/δ*) were expressed at lower levels in FGR placentas than in controls (Figure S5b). The findings suggest that many of the genes associated with lipid metabolism are downregulated in FGR placentas, which may explain the abnormal changes observed in several lipid components. We found the concentration of multiple lipids to be increased in the DJ and L layers of FGR mouse placentas. We investigated whether energy supply was altered in FGR placentas by measuring the expression of ATP regulatory proteins. We recognized that the expression of CII was unaltered in the DJ layer of FGR placentas; however, the abundance of CV was significantly increased by 23.9% and 48.0% in the DJ or L layers of FGR placentas, respectively (Fig. 6g). These findings indicate that energy metabolism may be altered in the FGR placentas.

### Effect of maternal exercise on rapamycin-induced FGR in mice

**Fig. 7:**
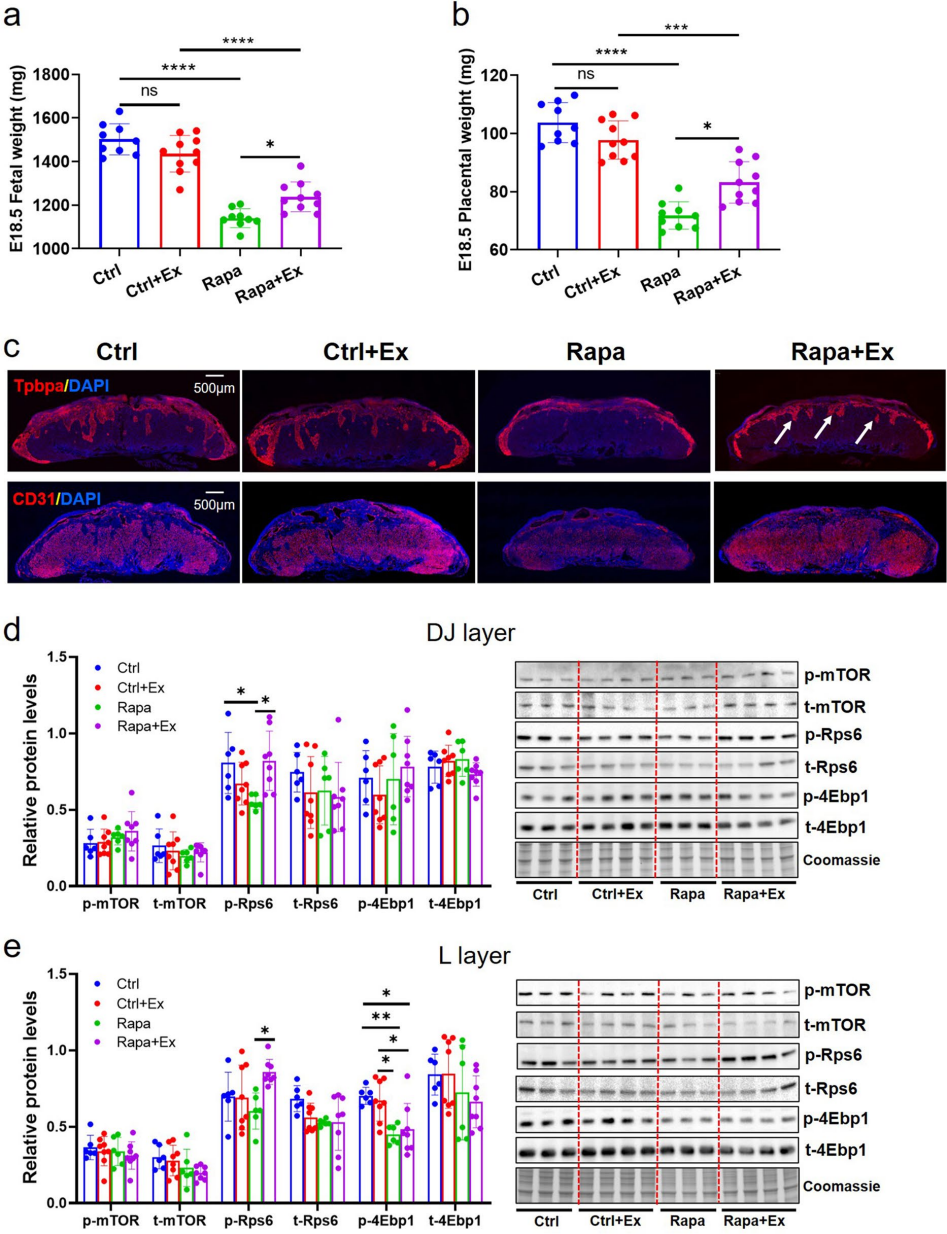
The effect of maternal exercise on fetal and placental weight, placental morphology and mTOR signaling in mouse FGR model caused by rapamycin (low-dose/ 0.01 mg Rapa). (**a**): The fetal weight at E18.5 in Ctrl (*n* =9 dams), Ctrl + Ex (*n* =10 dams), Rapa (*n* =9 dams) and Rapa + Ex group (*n* =10 dams). (**b**): The placental weight at E18.5 in Ctrl (*n* =9 dams), Ctrl + Ex (*n* =10 dams), Rapa (*n* =9 dams) and Rapa + Ex group (*n* =10 dams). (**c**): The IF staining of Tpbpa and CD31 in placentas from the four group (*n* =5/each group) at E18.5. The mTOR signaling activities in DJ-layer (**d**) and L-layer (**e**) of mouse placentas from Ctrl (*n* =6 dams), Ctrl + Ex (*n* =8 dams), Rapa (*n* =6 dams) and Rapa + Ex group (*n* =8 dams) at E18.5, respectively. One-way ANOVA test was used for the global comparison and Turkey’s test for the pairwise comparison, and *p*-value<0.05 was significant. ns: not significant, *p<0.05, **p<0.01, ***p<0.001, ****p<0.0001

As FGR placentas exhibited smaller size, high lipid state, and down-regulation of genes associated with lipid transport and metabolism, we speculated that high lipid content and decreased lipid transfer and supply may be because of the limited blood-exchange area in the labyrinth layer of FGR placentas. Studies report that physical exercise during pregnancy is beneficial to placental perfusion and fetal growth (Genest et al. 2012; Brett et al. 2015). We suggest that maternal exercise may promote lipid utilization in FGR placentas and therefore mitigate FGR induced by rapamycin. The experimental design diagram was shown in Figure S6 and consisted of control groups with or without exercise (Ctrl and Ctrl + Ex), and Rapa group with or without exercise (Rapa and Rapa + Ex). The data demonstrated that maternal exercise contributed to a significant increase in fetal and placental weight at late gestation (E18.5) in FGR pregnancies, although it did not fully reverse the reduced fetal growth (Fig. 7a) or placental weight (Fig. 7b). Additionally, we observed increased invasion of spongiotrophoblast cells, as evidenced by Tpbpa staining, in the labyrinth of FGR placentas exposed to maternal exercise (Fig. 7c and Figure S6a). IF staining revealed that CD31 intensity was decreased in FGR placentas compared to controls at E18.5, but increased with receiving maternal exercise (Fig. 7c and Figure S6b).

We investigated the effect of maternal exercise on the mTOR signaling pathway in rapamycin-induced FGR mouse placentas. As presented in Fig. 7d, the phosphorylation of Rps6 (p-Rps6) in the DJ layer was significantly decreased in the FGR group compared to controls, but was restored with maternal exercise exposure. In the L layer, we observed that placentas from pregnancies exposed to maternal exercise had an elevated p-Rps6 level, despite no significant difference seen between the unexercised FGR and control groups at E18.5 (Fig. 7e). Additionally, we found the phosphorylation of 4Ebp1 in the L layer of mouse placentas to be significantly reduced in the FGR group, regardless of maternal exercise, compared to the control group (Fig. 7e).

**Fig. 8:**
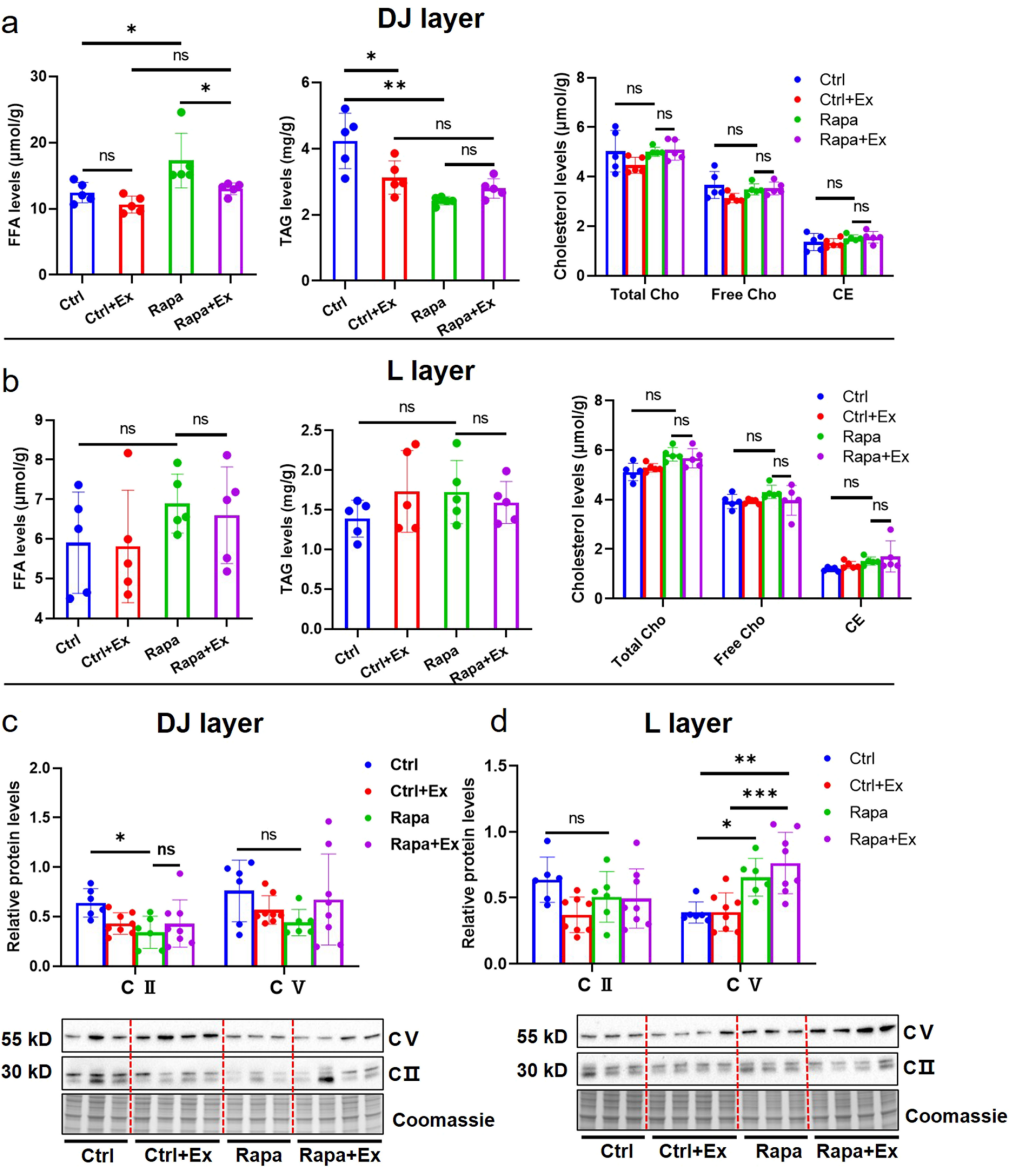
The change of lipid concentrations and ATP-associated protein levels in mouse placentas at E18.5. (**a**): The levels of FFA, TAG, total Cho, free Cho and CE in DJ-layer of mouse placentas from Ctrl, Ctrl+Ex, Rapa and Rapa+Ex group (*n* =5 dams/each group), respectively. (**b**): The levels of FFA, TAG, total Cho, free Cho and CE in L-layer of mouse placentas from Ctrl, Ctrl+Ex, Rapa and Rapa+Ex group (*n*=5 dams/each group), respectively. The expressions of ATP regulatory proteins (CII and CV) in DJ-layer (**c**) and L-layer (**d**) of mouse placentas from Ctrl (*n*=6 dams), Ctrl+Ex (*n*=8 dams), Rapa (*n*=6 dams) and Rapa+Ex group (*n*=8 dams), respectively. One-way ANOVA test was used for the global comparison and Turkey’s test for the pairwise comparison, and *p*-value<0.05 was significant. ns: not significant, *p<0.05, **p< 0.01, ***p< 0.001

Due to the changes in mTOR signaling observed in different placental regions of FGR mice exposed to maternal exercise, we supposed that placental lipid metabolism may also be affected. We tested the three lipids FFA, TAG, and Cho in mouse placentas. We found that, similar to E14.5, at E18.5 FGR placentas had increased FAA levels in the DJ layer compared with controls, but maternal exercise exposure lowered this level in FGR placentas closer to that of controls (Fig. 8a). When examining TAG levels in the DJ layer, in contrast to E14.5 when similar levels were observed in FGR and control placentas, at E18.5 decreased TAG levels were identified in FGR placentas and maternal exercise was not found to alter these levels (Fig. 8a). Total and free Cho as well as CE were measured and showed no significant differences in the DJ layer of placentas among the four groups at E18.5 (Fig. 8a). Unexpectedly, we did not find the levels of FFA, TAG, or Cho to be different in the L layer of FGR and control placentas with or without maternal exercise at E18.5 (Fig. 8b).

Finally, we tested whether maternal exercise altered the expression of ATP regulatory proteins. In the DJ layer of FGR placentas at E18.5 we found that CII expression was significantly decreased and not reversed by maternal exercise, whereas CV expression was not found to be different between the four groups (Fig. 8c). In the L layer of FGR placentas at E18.5 we found that CII expression was not changed but CV level was increased and maternal exercise seemed to further increase CV expression (Fig. 8d).

These results suggest that maternal exercise is beneficial for improving the rapamycin-induced FGR outcomes, and it may be associated with increased placental Rps6 activity as well as availability of FFA in the DJ layer.

## Discussion

The placenta is a complex organ with different structural subregions consisting of various cell types including syncytiotrophoblasts, cytotrophoblasts, mesenchymal cells, and fetal vascular cells (Wang and Zhao 2010). Different anatomic placental areas could play varying roles in normal fetal growth and development. Our previous study revealed that intrauterine injection of lipopolysaccharide resulted in different mTORC1 activities in different anatomic locations of the mouse placenta (Dong et al. 2019). These findings suggest that it is necessary to distinguish placental regions when analyzing molecular expressions and functional changes. In this study, we identified increased mTOR activity in fetal-side compared to maternal-side of human placentas. Similarly, in mouse placentas the key molecules of mTOR signaling exhibited different expression levels and phosphorylation activity in different anatomical regions (DJ layer vs L layer). A study by Napso et al. (2022) reported that the change in respiratory capacity differed in different placental regions under the condition of maternal diet-induced obesity, suggesting that the junctional and labyrinth layers have different functionality and response features.

FGR is a common complication of pregnancy and can have significant effects on long term neonatal outcomes Although a growing number of studies support mTOR signaling inhibition as an underlying mechanism of FGR (Gupta and Jansson 2019; Larqué et al. 2013; Roos et al. 2009), there remains inconsistencies in the literature, likely owing to differences in study methodology (Dong et al. 2020). Therefore, our study aimed to clarify the precise mechanism of placental mTOR activity in FGR.

We investigated the placental mTOR signaling pathway in full-term human SGA and AGA pregnancies. In contrast to previously published studies (Hung et al. 2017; Roos et al. 2007; Zhang et al. 2017), we found increased p-4EBP1 level but decreased t-mTOR level in the maternal-side of human SGA placentas, as well as reduced p-RPS6 level in fetal-side of SGA placentas. The variation in these results compared to previously published findings is thought to be due to optimization of detection methods in our study. For example, we tested mTOR-related molecules in the maternal- and fetal-side of placentas. In addition, we utilized Coomassie staining as the total protein reference used for calculating the relative values of protein bands in WB, which may be superior to the housekeeping proteins in terms of quantitative analysis (Welinder and Ekblad 2011).

Many different FGR models exist, including folate deficiency, maternal diet restriction, and hypoxia. As summarized in our review (Dong et al. 2020), studies of these models have identified the commonality of placental mTOR signaling pathway inhibition in FGR, indicating that it may be a key regulatory hub in the development of growth restriction. Based on this, we assume that inhibiting placental mTOR activity with rapamycin to induce FGR is a valid model to study the mechanism underlying FGR, especially the type without specific pathogenic factors. In 2021, Shao et al. (2021) developed the rapamycin-induced FGR mouse model by intraperitoneally injecting rapamycin at the dose of 1 mg/kg/day (approximately 0.04 mg/day per dam) during E10.5 to E13.5, and found the phosphorylation activities of mTOR and S6k (an upstream of Rps6) were significantly reduced in the bulk placentas from FGR group. In this study, we utilized the FGR model induced by a low-dose rapamycin (0.01 mg/day), we observed that the decreased mTOR activity occurred in the DJ layer of mouse FGR placentas. Our data demonstrate that low-dose rapamycin is associated with decreased intrauterine fetal growth and that mTOR signaling is affected to a greater extent in the DJ layer than the L layer of mouse placentas.

To understand the potential mechanism responsible for FGR due to the decreased placental mTOR signaling, we analyzed the morphology of mouse placentas and found that rapamycin-induced FGR placentas demonstrated reduced placental size and thickness, decreased ratio of junctional layer, and decreased vascular density. These morphological changes may be associated with abnormal placental structure and function. By the methods of whole placenta transcriptomics, we characterized that lipid metabolism may be affected in FGR placentas based on the enrichment analysis and expression identification of the DE mRNAs (*Enpp7*, *Acacb* and *Clps*). In addition, lipidomic examination of the DJ and L layers of mouse placentas, identified the increased levels of multiple lipid components linked with energy supply (such as FFA and TAG) in rapamycin-induced FGR placentas. However, other lipids (such as CL, PE, and PS) involved in cellular structure assembly were significantly reduced in FGR placentas, which may explain their reduced placental size. When analyzing the expression of genes related to lipid metabolism, despite the differential expression between the DJ and L layers, most of the relevant genes demonstrated reduced expression levels in FGR placentas compared to controls. As mTOR signaling plays a fundamental role in regulating lipid biosynthesis and metabolism, the observed changes in lipid molecules and corresponding regulatory genes could be attributed to mTOR inhibition in these FGR placentas (Caron et al. 2015). With the exclusion of the potential effect of maternal blood lipids on placental lipid levels, we suppose that the increased major lipids (FFA, CE and TAG) are due to suppressed lipid metabolism (including lipid oxidation, synthesis, and transport) in rapamycin-induced FGR mouse placentas. Importantly, we observed that mitochondrial respiratory chain (MRC) complexes V (CV, also called ATP synthase) were significantly increased in the L layer of FGR placentas compared to controls. These findings indicate that, as a consequence of altered lipid metabolism, there may be a compensatory increase in energy supply in FGR placentas to maintain fetal growth.

Strategies to prevent unexplained FGR are critical but limited in obstetric management. At present, effective interventions to reduce the occurrence of FGR mainly emphasize maintaining a healthy lifestyle (ex. avoiding tobacco and alcohol) and routine ultrasound monitoring (Berghella 2007). Additionally, some studies reported that special diets during pregnancy (like Mediterranean diet, omega 3 or folate supplementation) were associated with reducing FGR risk or promoting fetal growth (Crovetto et al. 2021; Vafai et al. 2023; Barchitta et al. 2020). However, there remains a significant need for finding an effective and acceptable method to prevent FGR. Since rapamycin-induced FGR placentas had increased lipid accumulation, and may have decreased utilization, we explored the effect of maternal exercise on fetal weight in the FGR model. Our data showed that maternal exercise in rapamycin-treated pregnancies, conducted during E0.5 to E16.5, increased partial fetal and placental weight. Maternal exercise was also associated with improved placental morphology in the FGR model and increased phosphorylation activity of Rps6 in the DJ and L layers of FGR placentas. Additionally, we observed that the FFA level in the DJ layer of FGR placentas returned to normal level with maternal exercise. These results suggest that maternal exercise improves placental insufficiency and promotes FFA metabolism in rapamycin-induced FGR, which can partially mitigate the development of FGR. In fact, some researchers have reported that pregnant women who participate in moderate physical activity have a low risk of low-birth weight babies (Xi et al. 2020; Walasik et al. 2020; Gollenberg et al. 2011; Takito and Benício 2010). However, other studies found no effect of maternal physical activity in reducing the risk for SGA neonates (Pastorino et al. 2019; Beetham et al. 2019; Davenport et al. 2018), with some actually reporting that vigorous physical activity may increase the risk (Legesse et al. 2020; Ehrlich et al. 2020). These discrepancies may be related to differences in exercise definition and study design. Currently, there is no strong evidence that moderate exercise during pregnancy is detrimental. Further research on maternal exercise is important and may assist in the development of recommendations for clinical practice to improve pregnancy outcomes, including in cases of FGR.

Although we found that maternal exercise mitigated some of the abnormal phenotypes in fetuses and placentas in the rapamycin-induced FGR mouse model, we cannot be certain that maternal activity directly regulates placental mTOR signaling or promotes lipid utilization and energy supply to the fetuses. The placenta has the ability to adapt to intrinsic and extrinsic environmental abnormalities in order to support fetal growth (Sandovici et al. 2012; Myatt 2006). We found that, compared to controls, p-mTOR level was decreased in the DJ layer of FGR placentas at E14.5, but showed no difference between the FGR and control groups at E18.5. In the L layer, the levels of FFA, TAG, and Cho were similar at E18.5 between the FGR and control groups, despite an increase identified at E14.5. Additionally, the increased CV expression in the L layer of FGR placentas at E14.5 was found to be also increased at E18.5 under the treatment of maternal exercise. Placental junctional and labyrinth layers support intrauterine fetal growth. The junctional layer provides the main endocrine function of the placenta by generating hormones, growth factors, and cytokines while labyrinth layer transports nutrients and gases from mother to fetus (Woods et al. 2018). Previous studies have identified the junctional layer structure as being particularly affected in cases of FGR, much more so than the labyrinth layer (Tao et al. 2022; Gualdoni et al. 2021; Roberts et al. 2021). We speculate that the junctional layer of the placenta may be more sensitive to insults associated with FGR compared to the labyrinth layer, and the labyrinth layer may primarily act as an adaptive regulator to sustain fetal growth.

It is important to note that there are also some limitations in this study. First, evidence reported by Tshering et al. (2021) showed that rapamycin can transfer to the fetal body through the placenta, and it may simultaneously restrict placental and fetal growth despite the controversial findings (Chu et al. 2008; Tshering et al. 2021; Ebrahimi-Fakhari et al. 2021). Additionally, the inhibition of placental mTOR activity is common in FGR caused by specific (like maternal malnutrition, pre-eclampsia, chemical exposures, etc.) or unknown factors. Therefore, the rapamycin-induced FGR model may be appropriate to simulate clinical FGR appeared in women who take sirolimus during pregnancy (Tshering et al. 2021; Hennig et al. 2017), or mimic FGR with unknown pathogenic factors. Second, although we found that mTOR signaling were changed in different anatomic regions of human and mouse placentas, from a detailed perspective, the changed molecular markers were not fully consistent between human and mouse placentas. We speculated there may exist different adaptive responsive mechanisms in FGR placentas between the two species. Moreover, despite the benefit of maternal exercise in mitigating mouse FGR, the clinical significance of maternal exercise in preventing FGR is required to be further proven.

## Conclusions

In summary, we have demonstrated that human and mouse placentas have different mTOR signaling activities in different anatomic regions in normal and FGR pregnancies. By utilizing the rapamycin-induced FGR mouse model, we revealed that the inhibition of mTOR signaling in DJ layer of mouse placenta leads to abnormal lipid metabolism appeared in the whole placenta, which may be involved in the occurrence of FGR. In addition, we found that maternal exercise is associated with improved placental mTOR activity and lipid metabolism, as well as increased fetal and placental weight in our FGR model. Our data indicate that maternal exercise could be an effective method to reduce the risk of FGR. Future studies are needed to expand upon these findings in order to improve pregnancy outcomes.

## Supplementary Information

Below is the link to the electronic supplementary material.

Supplementary file1 (XLSX 108 KB)

Supplementary file2 (XLSX 153 KB)

Supplementary file3 (DOCX 4246 KB)

## Data Availability

According to our department regulations, the original data of lipidomics and RNA sequencing data will be shared via e-mail from corresponding author on reasonable request.

## Abbreviations

AGA: Appropriate-for-gestational-age
ATP: Adenosine triphosphate
BMP: Bis-monoacylglycerol phosphate
CE: Cholesteryl esters
Cho: Cholesterols
CL: Cardiolipins
DAG: Diacylglycerols
DJ: Decidua junction
DMSO: Dimethyl sulfoxide
FFA: Free fatty acid
FGR: Fetal growth restriction
Gb3: Ceramide trihexoside
GO: Gene ontology
HE: Hematoxylin & eosin
IF: Immunofluorescence
KEGG: Kyoto encyclopedia of genes and genomes
L: Labyrinth
LPE: Lyso- phosphatidylethanolamines
LPS: Lysophosphatidylserines
mTOR: Mechanistic target of rapamycin
PBS: Phosphate-buffered saline
PC: Phosphatidylcholines
PE: Phosphatidylethanolamines
PS: Phosphatidylserines
RT-qPCR: Real-time quantitative polymerase chain reaction
SGA: Small-for-gestational-age
TAG: Triacylglycerols
WB: Western blot
