# Supplementary material for: Fetal growth restriction exhibits various mTOR signaling in different regions of mouse placentas with altered lipid metabolism
Source: Cell Biol Toxicol. 2024 Mar 7;40(1):15. doi: 10.1007/s10565-024-09855-8 (PMC10920423; doi:10.1007/s10565-024-09855-8)
Supplement: Supplementary file 3 — Supplementary file3 (DOCX 4246 KB) [file 10565_2024_9855_MOESM3_ESM.docx]

Supplementary Table 1. Clinical characteristics of full-term normal pregnancies

|  | Normal pregnancies (n=12) |
| --- | --- |
| Maternal age (years) | 29.9±3.5 |
| Gestational age (days) | 271.0±4.7 |
| Maternal BMI (Kg/m^2^) | 28.7±3.5 |
| Systolic pressure (mmHg) | 111.2±10. 9 |
| Diastolic pressure (mmHg) | 74.7±6.7 |
| Gravidity  1  >1 | 4  8 |
| Newborn sex  female  male | 6  6 |
| Birth weight (g) | 3334.6±127.2 |
| Placental weight (g) | 580.0±81.7 |

Continuous variables were shown as mean±standard deviation, counting data described as frequency. Maternal BMI was calculated in women who were about to give birth.

Supplementary Table 2. The baseline clinical data of full-term AGA and SGA pregnancies

|  | AGA (n=10) | SGA (n=8) | *p* value |
| --- | --- | --- | --- |
| Maternal age (years) | 29.2±3.0 | 28.5±3.5 | 0.655 |
| Gestational age (days) | 270.8±5.0 | 270.1±3.2 | 0.746 |
| Maternal BMI (Kg/m^2^) | 28.98±3.1 | 27.8±4.4 | 0.500 |
| Systolic pressure (mmHg) | 109.3±9.9 | 106.8±8.6 | 0.573 |
| Diastolic pressure (mmHg) | 74.6±7.1 | 71.13±2.3 | 0.202 |
| Gravidity  1  >1 | 3  7 | 4  4 | 0.748 |
| Newborn sex  female  male | 4  6 | 4  4 | 0.671 |
| Birth weight (g) | 3341.5±131.2 | 2762.5±87.6 | 0.000 |
| Placental weight (g) | 583.0±80.6 | 515.6±86.4 | 0.107 |

Continuous variables were shown as mean±standard deviation, counting data described as frequency. Maternal BMI was calculated in women who were about to give birth.

Supplementary Table 3 The overlapped lipid components with differential concentrations between DJ and L side of mouse placentas from Ctrl and Rapa group

|  | DJ side | | | L side | | |
| --- | --- | --- | --- | --- | --- | --- |
| Lipid name | Fold change  (Rapa/Ctrl) | change | *p* value | Fold change  (Rapa/Ctrl) | change | *p* value |
| BMP36:2(18:2_18:0) | 1.25 | up | 0.03 | 1.46 | up | 0.04 |
| CE-16:0 | 1.65 | up | 0.04 | 1.74 | up | 0.00 |
| CE-18:0 | 1.74 | up | 0.00 | 2.05 | up | 0.00 |
| CE-18:2 | 1.85 | up | 0.01 | 1.84 | up | 0.00 |
| CE-18:3 | 1.92 | up | 0.00 | 1.74 | up | 0.01 |
| CE-20:3 | 1.75 | up | 0.00 | 1.84 | up | 0.00 |
| CE-20:4 | 1.92 | up | 0.00 | 1.63 | up | 0.02 |
| CE-20:5 | 2.17 | up | 0.00 | 2.09 | up | 0.00 |
| CE-22:4 | 1.66 | up | 0.03 | 1.41 | up | 0.03 |
| CE-22:6 | 1.91 | up | 0.01 | 2.03 | up | 0.00 |
| CL66:4(16:1) | 0.25 | down | 0.00 | 0.28 | down | 0.00 |
| CL68:5(16:1) | 0.34 | down | 0.00 | 0.39 | down | 0.00 |
| CL68:5(18:2) | 0.64 | down | 0.00 | 0.63 | down | 0.00 |
| CL68:6(16:1) | 0.41 | down | 0.00 | 0.44 | down | 0.00 |
| CL70:4(16:1) | 0.51 | down | 0.00 | 0.54 | down | 0.00 |
| CL70:5(16:1) | 0.55 | down | 0.00 | 0.58 | down | 0.00 |
| CL70:5(18:2) | 0.80 | down | 0.01 | 0.85 | down | 0.01 |
| CL70:6(16:1) | 0.68 | down | 0.00 | 0.68 | down | 0.00 |
| CL70:6(18:2) | 0.78 | down | 0.01 | 0.81 | down | 0.00 |
| CL70:7(16:1) | 0.76 | down | 0.01 | 0.84 | down | 0.03 |
| CL72:8(16:1) | 0.75 | down | 0.02 | 0.73 | down | 0.00 |
| CL72:9(16:1) | 0.75 | down | 0.00 | 0.79 | down | 0.00 |
| CL74:10(16:1) | 0.69 | down | 0.02 | 0.66 | down | 0.00 |
| CL74:7(18:2) | 1.39 | up | 0.03 | 1.21 | up | 0.00 |
| CL74:8(18:2) | 1.32 | up | 0.02 | 1.27 | up | 0.00 |
| CL74:9(18:2) | 1.28 | up | 0.00 | 1.24 | up | 0.00 |
| CL74:9(20:3) | 1.24 | up | 0.04 | 1.34 | up | 0.00 |
| CL76:10(18:2) | 1.37 | up | 0.02 | 1.14 | up | 0.02 |
| CL76:11(16:1) | 0.66 | down | 0.01 | 0.71 | down | 0.00 |
| CL76:13(16:1) | 0.58 | down | 0.00 | 0.56 | down | 0.00 |
| CL76:14(16:1) | 0.59 | down | 0.01 | 0.55 | down | 0.00 |
| CL78:12(18:2) | 1.60 | up | 0.03 | 1.25 | up | 0.03 |
| CL78:13(18:2) | 1.33 | up | 0.02 | 1.32 | up | 0.02 |
| DAG38:4(18:0/20:4) | 1.30 | up | 0.04 | 1.19 | up | 0.03 |
| DAG38:5(18:0/20:5) | 1.30 | up | 0.04 | 1.38 | up | 0.00 |
| DAG38:6(16:0/22:6) | 1.50 | up | 0.01 | 1.28 | up | 0.01 |
| FFA18:2 | 1.50 | up | 0.00 | 1.50 | up | 0.01 |
| FFA20:3 | 1.48 | up | 0.01 | 1.52 | up | 0.04 |
| FFA20:4 | 1.84 | up | 0.01 | 1.59 | up | 0.02 |
| FFA20:5 | 2.12 | up | 0.01 | 1.95 | up | 0.02 |
| FFA22:6 | 1.89 | up | 0.00 | 1.52 | up | 0.01 |
| Gb3 d18:1/16:0 | 1.45 | up | 0.00 | 1.27 | up | 0.04 |
| LPE18:0p | 1.24 | up | 0.04 | 1.25 | up | 0.03 |
| LPE18:1 | 0.55 | down | 0.04 | 0.72 | down | 0.01 |
| LPS16:0 | 0.62 | down | 0.02 | 0.73 | down | 0.01 |
| LPS18:1 | 0.79 | down | 0.02 | 0.73 | down | 0.03 |
| PC40:6 | 1.69 | up | 0.02 | 1.23 | up | 0.01 |
| PE34:1 | 0.48 | down | 0.00 | 0.65 | down | 0.00 |
| PE36:1 | 0.66 | down | 0.00 | 0.68 | down | 0.01 |
| PE38:2p | 0.62 | down | 0.00 | 0.78 | down | 0.00 |
| PS 32:0 | 0.72 | down | 0.02 | 0.68 | down | 0.02 |
| PS 32:1 | 0.48 | down | 0.00 | 0.70 | down | 0.02 |
| PS 34:1 | 0.61 | down | 0.00 | 0.73 | down | 0.03 |
| PS 40:4 | 0.91 | down | 0.02 | 0.86 | down | 0.03 |
| PS 40:6 | 1.30 | up | 0.03 | 1.26 | up | 0.00 |
| TAG48:0(16:0) | 1.48 | up | 0.04 | 1.36 | up | 0.04 |
| TAG50:2(16:0) | 1.35 | up | 0.04 | 1.51 | up | 0.00 |
| TAG50:2(18:2) | 1.58 | up | 0.02 | 1.50 | up | 0.00 |
| 16:1-carnitine | 0.52 | down | 0.02 | 0.56 | down | 0.00 |
| 18:1-carnitine | 0.55 | down | 0.01 | 0.60 | down | 0.00 |
| 20:1-carnitine | 0.43 | down | 0.00 | 0.54 | down | 0.04 |

BMP: bis-monoacylglycerol phosphate, CE: cholesteryl esters, CL: cardiolipins, DAG: diacylglycerols, FFA: free fatty acid, Gb3: ceramide trihexoside, LPE: lyso- phosphatidylethanolamines, LPS: lyso-phosphatidylserines, PC: phosphatidylcholines; PE: phosphatidylethanolamines, PS: phosphatidylserines, TAG: triacylglycerols.

Supplementary Table 4 RT-qPCR primer sequences for mouse

| Gene | Forward primer（5’-3’） | Reverse primer（5’-3’） |
| --- | --- | --- |
| *Cpt1a*  *Cpt1b*  *Cpt1c*  *Acaca*  *Acacb*  *Fasn*  *Slc27a2*  *Slc27a4*  *Slc27a6*  *Cd36*  *Srebf1*  *Srebf2*  *Soat1*  *Soat2*  *Ppar-α*  *Ppar-β/δ*  *Ppar-γ*  *Enpp7*  *Clps*  *Cyp4f18*  *Msr1*  *Tpbpa*  *β-Actin* | CTACATCACCCCAACCCATATT  GAGGAAGGGTAGAGTGGGCAGAG  CATTGGTCAGAATCTTTTCCGG  CCCAGAGATGTTTCGGCAGTCAC  CCTTTGGCAACAAGCAAGGTA  TAAAGCATGACCTCGTGATGAA  CCCAGGATGTCATCTATACCAC  GGTTACCTGTACTTCCGAGATC  CTGGTCACGGTGCTGGATAAGTTC  CTTTGAAAGAACTCTTGTGGGG  GCTACCGGTCTTCTATCAATGA  TTTTACTGAAGTAGAGCGGGTC  CACGACTGGCTCTACTACTATG  CGAGATGCTGCGGTTTGGAGAC  GAGCTGCAAGATTCAGAAGAAG  CGAGTTTGCTGTCAAGTTCAAT  CCAAGAATACCAAAGTGCGATC  AAGCCCAGTATATGACTCCTGC  GAACAGTATGCAGTGTAAGAGCA  TGTTTTTGAGAACTCTCTTCGCC  GCACAATCTGTGATGATCGCT  TTCCTAGTCATCCTATGCCTGG  GAAATCGTGCGTGACATCAAAG | GATCCCAGAAGACGAATAGGTT  TCATCCAGGGTCACAAAGAAAGCAG  GTACAGACTCTAGGTACTTGCG  GTCAGGATGTCGGAAGGCAAAGG  AGTCGTACACATAGGTGGTCC  GAAGTTCAGTGAGGCGTAGTAG  CAATGTACTGAATGACCGTGAC  CCTTTTTCAAGGTCTGTGCAAA  AGCGAGGAGTGGTTCAGGAGAG  GTCTGTGCCATTAATCATGTCG  CGCAAGACAGCAGATTTATTCA  CATGCATGGCTCTACAGGTATA  CAAAGAACATGAAGAGCACGAA  AGGTGCGGTAGTAGTTGGAGAAGG  GAATCTTTCAGGTCGTGTTCAC  GAATTCTAGAGCCCGCAGAAT  TCACAAGCATGAACTCCATAGT  ACCGTGCTGGTGGTATTGTAG  GCAGATGCCATAGTTGGTGTTG  TGGAATATGCGGATGACTGGG  CCCAGCATCTTCTGAATGTGAA  GGTCATTTTCGCTACTGTGAAGT  TGTAGTTTCATGGATGCCACAG |


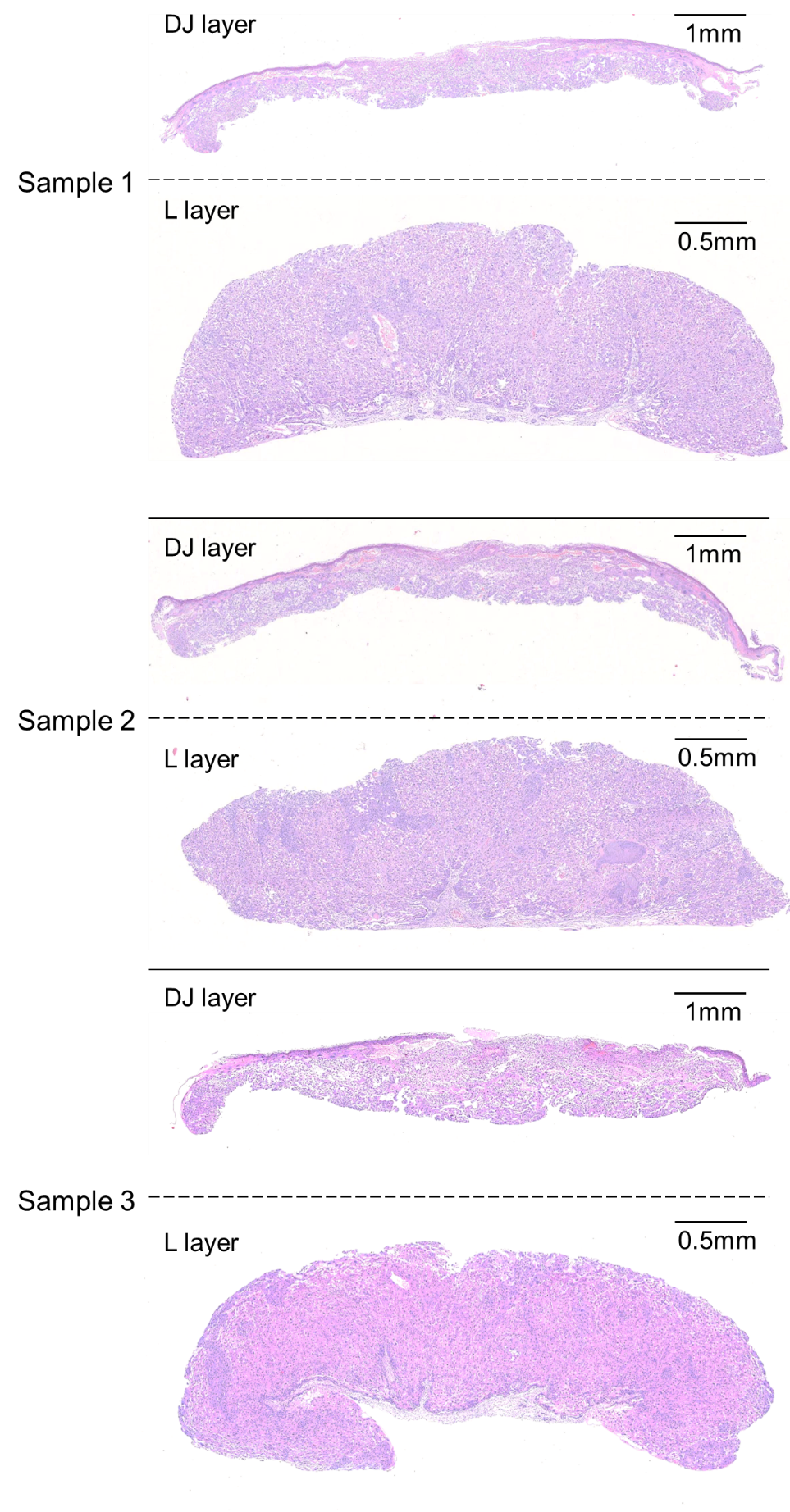


Figure S1 HE staining of DJ and L layers of placentas at E14.5 from three different pregnant mice.


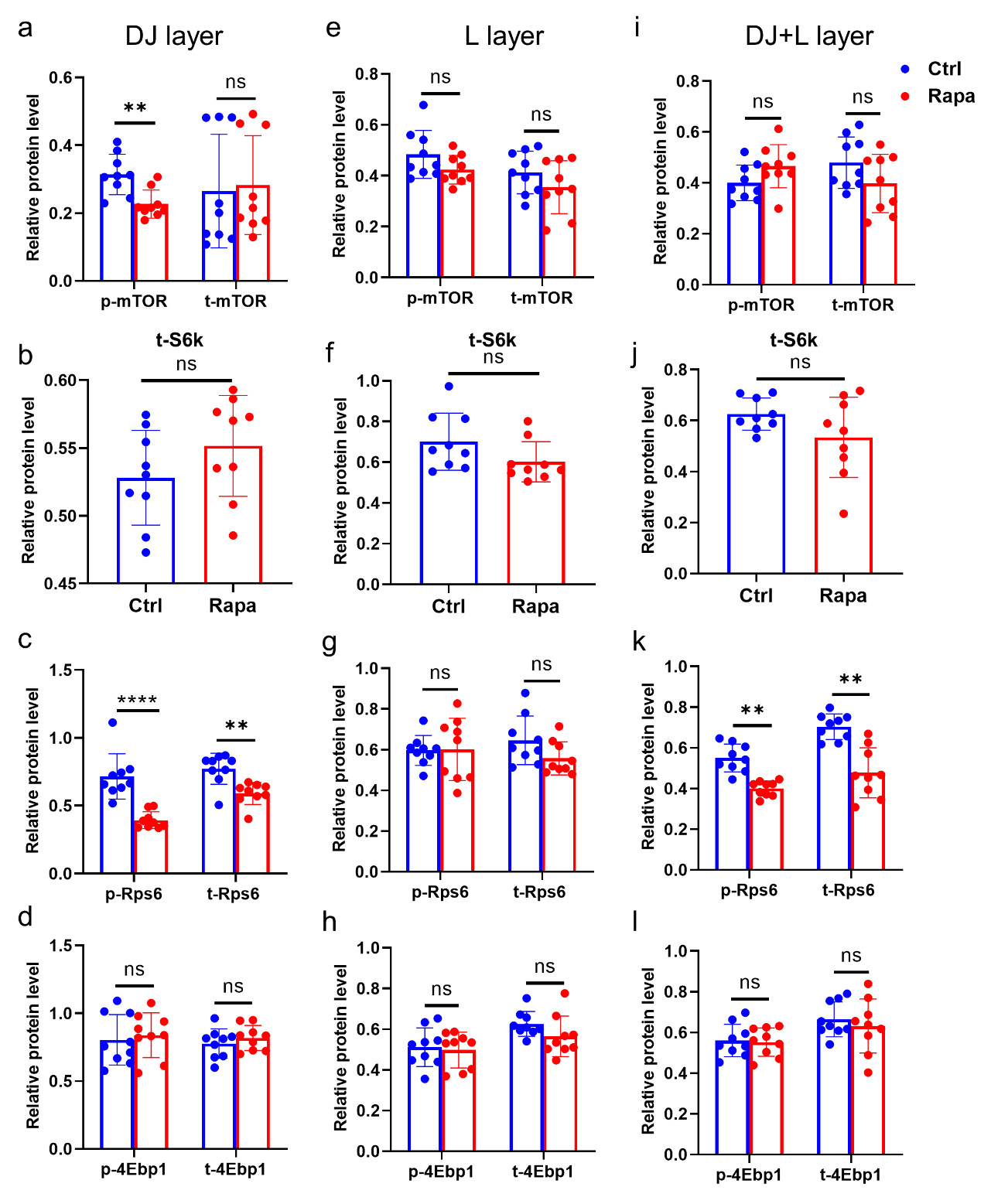


**Figure S2** The analysis of the key proteins of mTOR signaling pathway in mouse placentas between Ctrl and Rapa group (low-dose/ 0.01mg Rapa) at E14.5. (a-d): The expressions of mTOR-related proteins in placental DJ-layers between the two groups (n=9/each group). (e-h): The expressions of mTOR-related proteins in placental L-layers between the two groups (n=9/each group). (i-l): The expressions of mTOR-related proteins in placental DJ+L layers between the two groups (n=9/each group). Student’s t-test was used for statistical analysis and *p*-value <0.05 was significant (ns: not significant). ***p*<0.01, *****p*<0.0001.


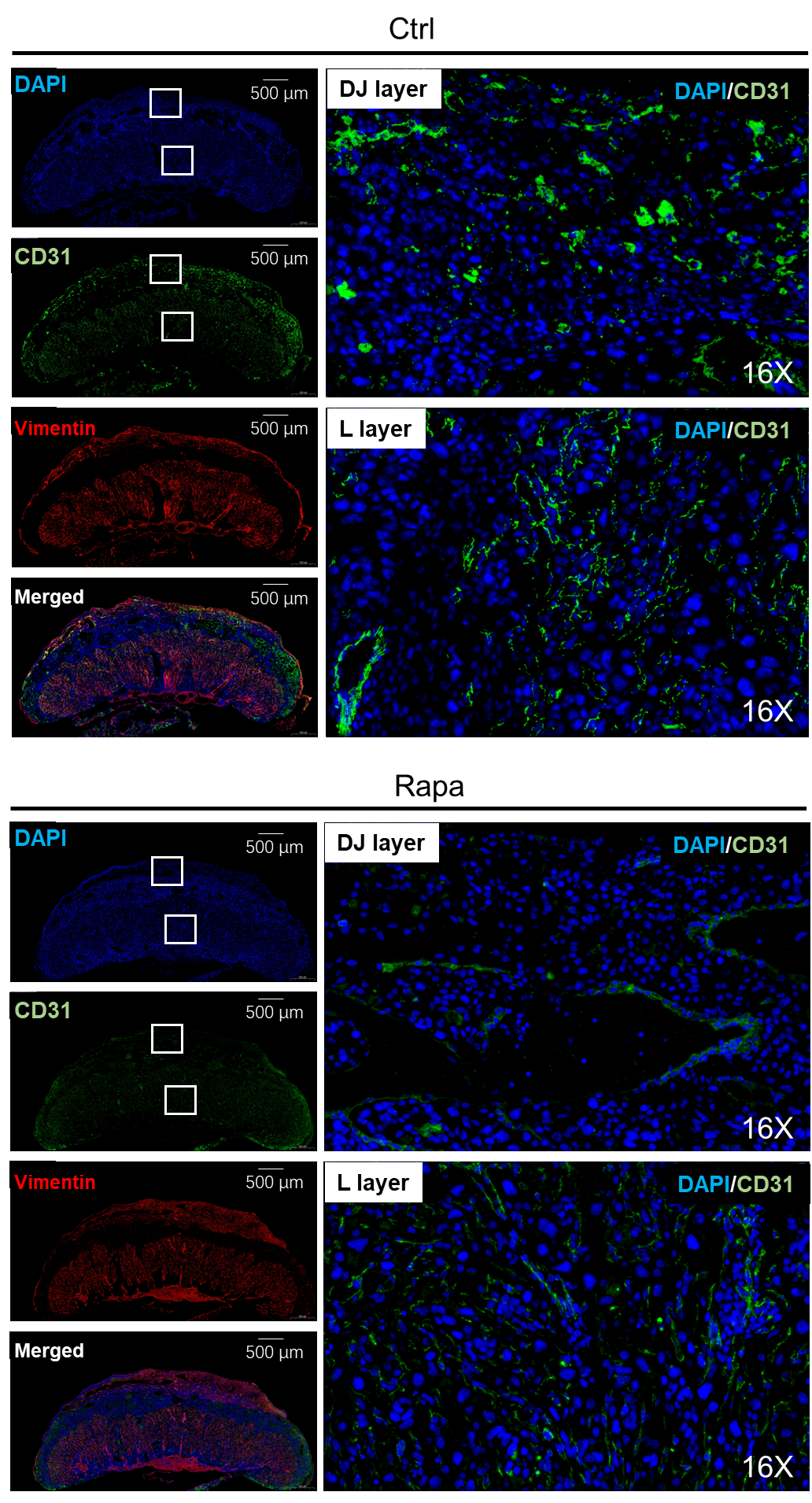


**Figure S3** The IF staining of CD31 and Vimentin with DAPI in E14.5 mouse placentas from Ctrl and Rapa groups. The magnified pictures of CD31 staining in DJ and L layers of mouse placentas were also shown here.


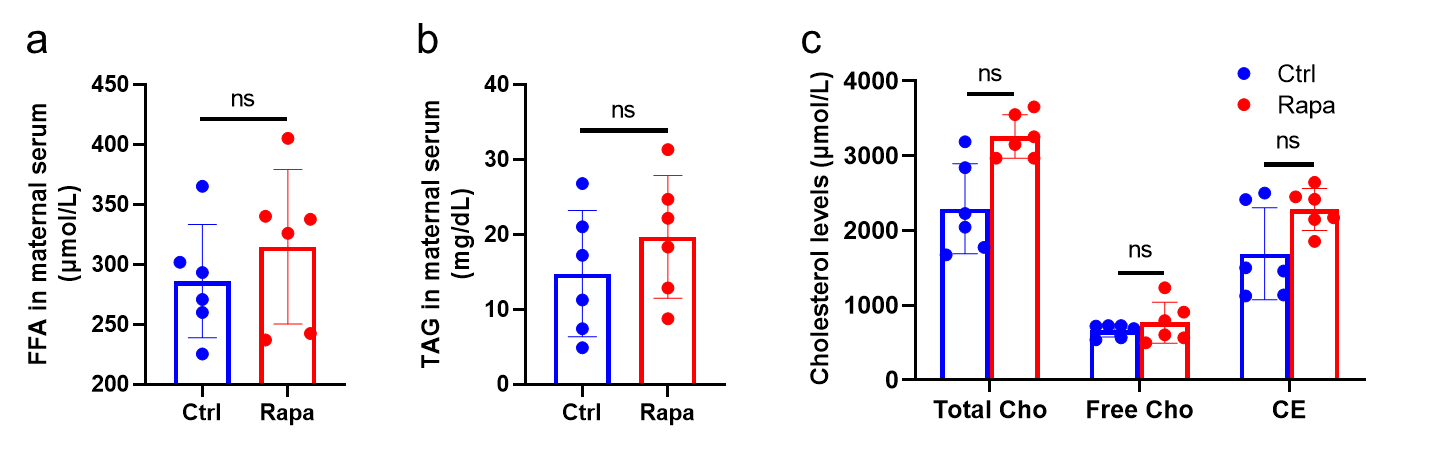


**Figure S4** The levels of FFA (a), TAG (b) and Cho (c) in peripheral serum of pregnant mice between Ctrl and Rapa group (n=6/each group) at E14.5. Student’s t-test was used for statistical analysis and *p*-value <0.05 was significant (ns: not significant).


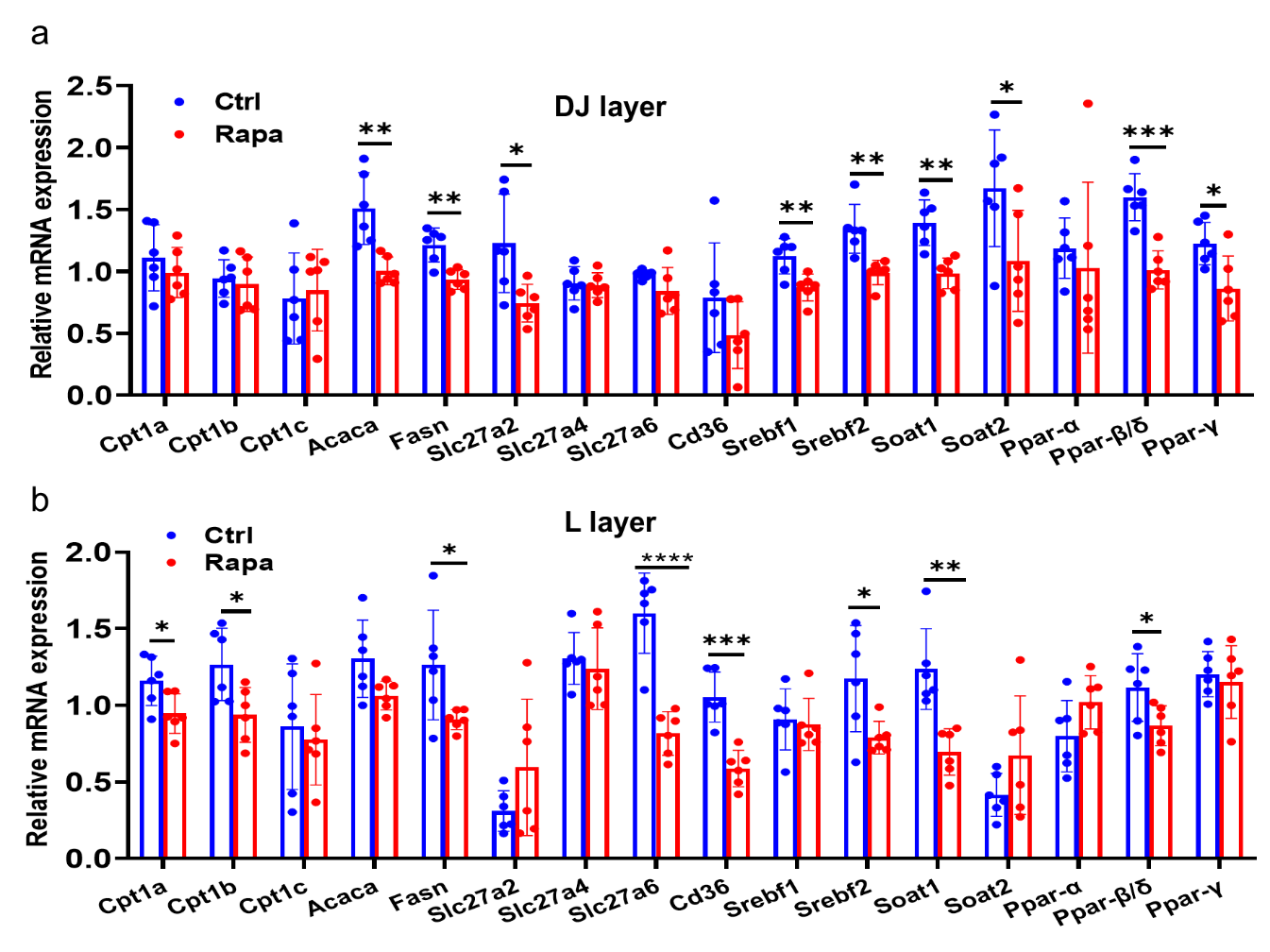


**Figure S5** The mRNA expressions of genes linked with lipid oxidation (*Cpt1a*, *Cpt1b*, *Cpt1c*, *Acaca*) and lipogenesis (Fasn), lipid transport (*Slc27a2*, *Slc27a4*, *Slc27a6* and *Cd36*) and relevant regulation factors (*Srebf1*, *Srebf2*, *Soat1*, *Soat2*, *Ppar-α*, *Ppar-β/δ* and *Ppar-γ*) in DJ-sides (a) or L-sides (b) of mouse placentas between Ctrl and Rapa group (low-dose/ 0.01mg Rapa) (n=6/each group) at E14.5, respectively. Student’s t-test was used for statistical analysis. **p*<0.05, ***p*<0.01, ****p*<0.001, *****p*<0.0001.


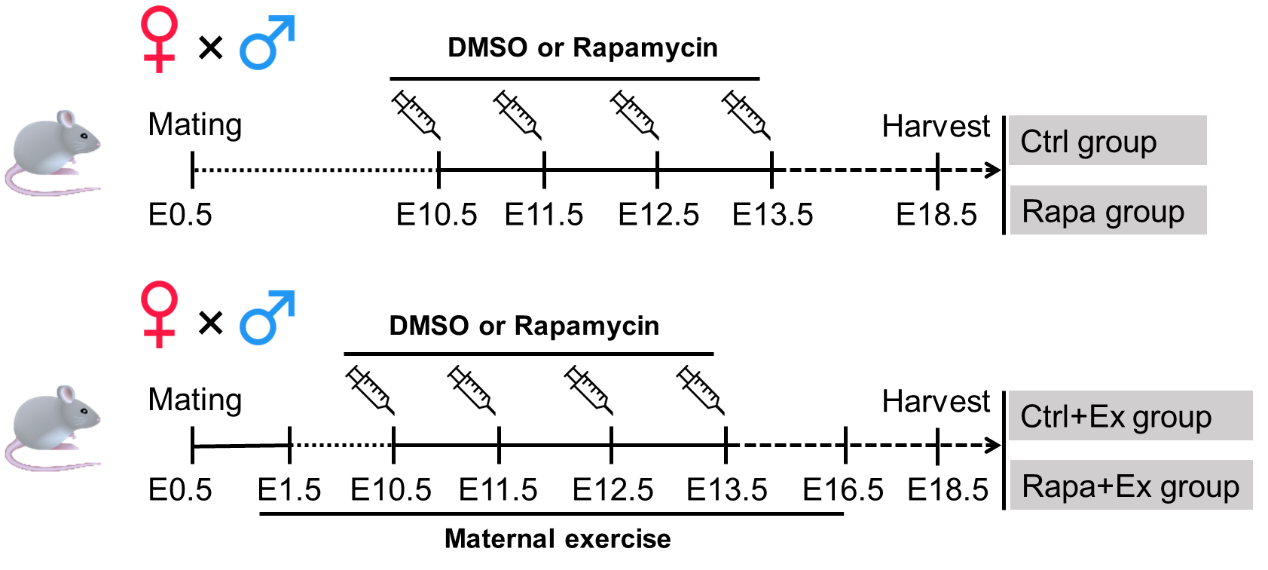


**Figure S6** The experimental design in exploring whether maternal exercise improves FGR induced by rapamycin. Pregnant mice were divided to four groups, including: a) Ctrl group treated with DMSO injection at E10.5-E13.5; b) Rapa treated with rapamycin (low-dose/ 0.01mg Rapa) injection at E10.5-E13.5; c) Ctrl+Ex group experiencing running exercise during E1.5-E16.5 and DMSO injection at E10.5-E13.5; d) Rapa+Ex group undergoing running exercise during E1.5-E16.5 and rapamycin injection (low-dose/ 0.01mg Rapa) at E10.5-E13.5.


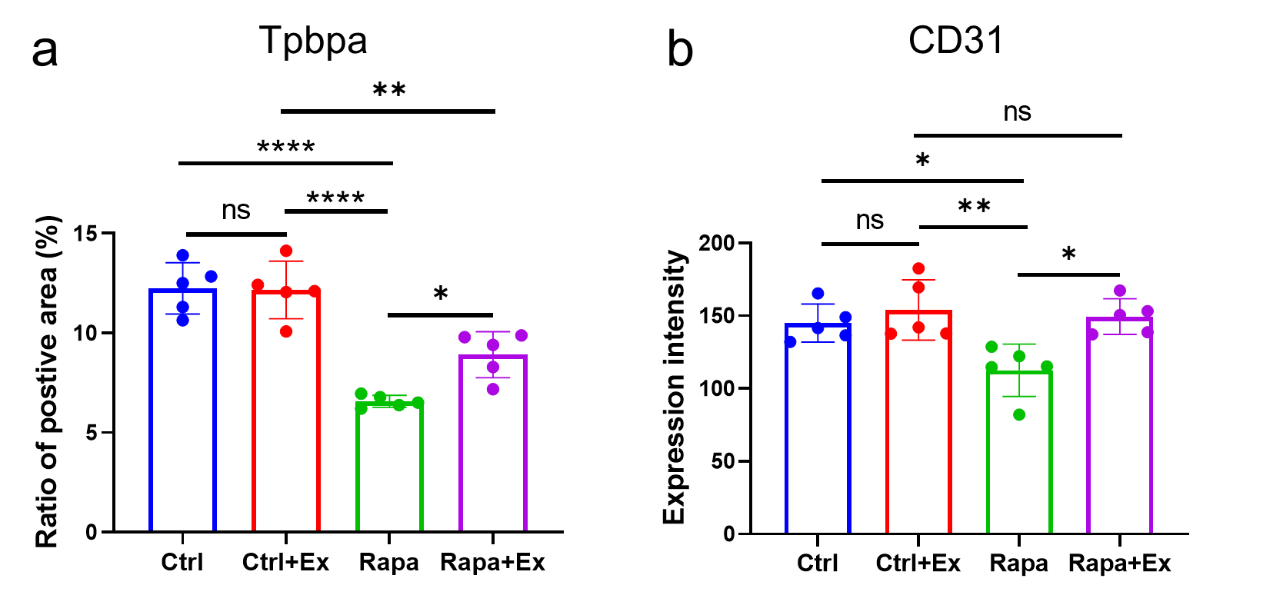


**Figure S6** The analysis of IF staining of Tpbpa (a) and CD31 (b) in placentas from Ctrl, Ctrl+Ex, Rapa, and Rapa+Ex groups (n=5/each group) at E18.5, respectively. ns: not significant, **p*<0.05, ***p*<0.01, ****p*<0.001, *****p*<0.0001.
